# Noncoding RNA Transcripts Based Molecular Phenotyping of MASLD and MASH Patients

**DOI:** 10.1002/lci2.70053

**Published:** 2026-07-16

**Authors:** Frida M. Delgadillo, Karla Perez, Barbara Yang, Subramanian Dhandayuthapani, Murali M. Yallapu, Subhash C. Chauhan, Enrique I. Ramos, Shrikanth S. Gadad

**Affiliations:** 1 Department of Biological Sciences, The University of Texas at El Paso, El Paso, Texas, USA; 2 Division of Cancer Immunology and Microbiology, Medicine and Oncology Integrated Service Unit, The University of Texas Rio Grande Valley School of Medicine, McAllen, Texas, USA; 3 South Texas Center of Excellence in Cancer Research, The University of Texas Rio Grande Valley School of Medicine, McAllen, Texas, USA

**Keywords:** long noncoding RNAs, MASH, MASLD, noncoding transcripts

## Abstract

**Background and Aims:**

Metabolic dysfunction-associated steatotic liver disease (MASLD) is a progressive hepatic metabolic disorder characterized by excessive fat accumulation in hepatocytes. MASLD can advance to metabolic dysfunction-associated steatohepatitis (MASH). These conditions often lead to hepatic fibrosis, cirrhosis and hepatocellular carcinoma (HCC) if not timely diagnosed and managed; thus, molecular phenotyping of MASLD and MASH conditions is highly desirable. The analyses were performed to identify distinct, differentially expressed genes, particularly noncoding ones, in MASLD and MASH patient samples, thereby expanding diagnostic and therapeutic options.

**Methods:**

We extracted the data from a recent transcriptomic study by Govaere et al. on MASLD and MASH patient samples. We focused on transcripts with limited coding potential to broaden the scope of biomarker or therapeutic target identification, given their very specific expression patterns.

**Results:**

This analysis identified long noncoding RNAs (lncRNAs) that are differentially expressed among MASLD and MASH conditions. Among the up-regulated lncRNAs, 49 were specific to MASLD, such as *MYCNOS*. In contrast, 88 were unique to MASH, such as *ELFN2-208*, *LINC01507* and *LINC01435*. Similarly, among the down-regulated noncoding transcripts, 93 were unique to MASLD, including *AADACL2-AS1* and *ST8SIA6-AS1*, whereas 132 were unique to MASH, such as *SYBU-AS1*, *LGALS17A-207, ZNF474-AS1* and *LINC01116*. Additionally, we employed the Genomic Regions Enrichment of Annotations Tool (GREAT) to assess their functional relevance in MASLD or MASH and to link them to biologically meaningful terms.

**Conclusion:**

This study broadens the scope of noncoding RNA-based MASLD/MASH biomarker and therapeutic target identification, which ultimately aids early diagnosis of these prominent liver diseases and helps prevent advanced liver diseases such as HCC.

## Lay Summary

The analysis of gene expression identifies unique and shared expressed genes between MASLD and MASH, focusing on long noncoding RNAs (lncRNAs) that are specific to each and those that overlap. Using lncRNAs' tissue-specific expression, disease progression can be monitored, aiding in the diagnosis of advanced liver disease.

## 1 ∣ Introduction

Non-alcoholic fatty liver disease (NAFLD) is a progressive metabolic disorder affecting the liver, recently reclassified as metabolic dysfunction-associated steatotic liver disease (MASLD) [1]. MASLD has become an epidemic, with studies projecting that about 33.5% of adults globally will be affected by this disease by 2030 [2]. In the United States, MASLD was reported to have the highest prevalence among Hispanic individuals, followed by White individuals, and the lowest prevalence in Black individuals [3]. Also, among the population affected with MASLD, the Hispanic population was at a higher risk of the disease progressing to non-alcoholic steatohepatitis (NASH) [3] and was recently renamed as metabolic dysfunction-associated steatohepatitis (MASH). MASLD can lead to MASH, fibrosis, cirrhosis and eventually hepatocellular carcinoma (HCC) [4]. MASLD is often asymptomatic and more prone to be diagnosed in people with obesity, type 2 diabetes and metabolic syndrome. There are four stages associated with MASLD: F0, which does not present fibrosis; F1, with perisinusoidal or portal fibrosis; F2, with sinusoidal or periportal fibrosis without bridging; F3, with bridging fibrosis; and F4, with cirrhosis [5]. MASLD is characterized by hepatic lipid accumulation, oxidative and endoplasmic reticulum stress, and lipotoxic damage [6]. Identifying genes that affect these processes at the transcriptomic level is crucial for identifying biomarkers and therapeutic targets that can address the development of MASLD.

Several studies have been conducted to understand how MASLD progresses and the pathways involved in its progression to HCC. In a study by Sveinbjornsson et al. [7], a genome-wide association study was conducted for MASLD, cirrhosis and HCC. They identified *MTARC1* and *GPAM* genes involved in lipid metabolism as potential targets when they have a loss of function, further establishing that metabolic and lipid-handling pathways contribute to MASLD progression [7]. Another multicentre transcriptomic analysis by Govaere et al. [8] identified a 25-gene signature associated with fibrosis and disease activity at the protein level (AKR1B10, GDF15). In a more recent study by the same group, they performed single-cell deconvolution, which identified probable hepatic cell types contributing to the proteomic changes; they identified four proteins (ADAMTSL2, AKR1B10, CFHR4 and TREM2) [9]. In these studies, these groups have identified potential therapeutic targets and demonstrated that large-scale transcriptomic profiling provides important insights into MASLD pathogenesis and progression.

However, these studies predominantly examine protein-coding genes; it is crucial to also investigate the noncoding regions of the genome that are transcribed into RNAs with limited or no coding capacity, and given their tissue and cell-specific expression patterns, they may provide novel, unique, specific biomarkers and therapeutic targets for MASLD. In the present study, we leveraged the whole-transcriptomic dataset generated by Govaere et al. [8], which is available for over 200 liver biopsy samples from patients diagnosed with MASLD or MASH, to identify differentially expressed transcripts compared with normal liver tissue. Our analyses focused explicitly on long noncoding RNAs (lncRNAs) due to their similarity with mRNAs, yet these are functionally distinct in driving various cellular functions [10-12]. Another characteristic feature of lncRNAs is their tight association with different biological processes, and their restricted expression patterns to specific cell types or tissues make them promising candidates as therapeutic targets or as biomarkers for monitoring disease progression, such as in MASLD. But unlike proteins, studies defining their roles are limited, which makes it challenging to perform gene ontology analyses [13]; hence, we used the Genomic Regions Enrichment of Annotations Tool (GREAT) to understand the functional relevance of these lncRNAs [14]. Collectively, this study identified differentially regulated lncRNAs expressed in MASLD and MASH and attempted to understand their significance in the context of disease pathogenesis. Our findings provide insights into predisposed conditions that could exacerbate disease progression and identify potential therapeutic targets.

## 2 ∣ Materials and Methods

### 2.1 ∣ Transcriptomic Data

The principal transcriptomic dataset used in this study was obtained from the National Center for Biotechnology Information resources, Gene Expression Omnibus (NCBI-GEO) under accession GSE135251. This dataset comprises RNA-seq data from 216 snap-frozen liver biopsies, including 10 control samples; the control samples consist of healthy obese individuals and 206 MASLD cases representing different fibrosis stages. The liver biopsy samples were obtained from 206 MASLD patients in France, Germany, Italy or the United Kingdom, and 10 healthy obese control cases without any biochemical or histological evidence of MASLD in France, as described in the original publication by Govaere et al. [8]. In our study, sample classification was divided into Control, MASLD and MASH groups based on the original dataset annotations. In the source study, they defined the presence of steatosis, lobular inflammation and hepatocellular ballooning. However, we did not reassign diagnostic categories; instead, we relied on the curated labels. We performed comparisons based on the group labels (Control, MASLD, MASH). Fibrosis stage was not included as a covariate in the differential expression of the models. Therefore, observed transcriptomic differences may partially reflect variation in fibrosis severity between groups. Also, while inflammation grade and hepatocellular ballooning scores were part of the histological assessment used in the original study, these variables were not independently incorporated into our analysis. Instead, we utilized the pre-defined group classifications.

### 2.2 ∣ Read Count Normalization

The raw sequencing reads (fastq files) and series matrix files were downloaded from the NCBI GEO repository. Read count normalization and differential gene expression analyses were calculated using the DESeq2 R package (differential gene expression analysis based on the negative binomial distribution, version 1.48.1) [15]. This normalization step involved adjusting raw read counts to account for technical biases across samples, such as differences in sequencing depth, to ensure that observed differences in gene expression are due to biological variation rather than technical artefacts [16, 17]. DESeq2 employs a method called ‘size factor’ normalization. It first calculates the median ratio of observed counts to the geometric mean across all samples for each gene [18]. This is calculated as a size factor that represents each sample's relative sequencing depth. The size factor is used to divide the raw read counts to generate normalized counts.

### 2.3 ∣ Annotation and Composition of Noncoding Genes

The gene list obtained after the DESeq2 analysis was queried in the R statistical software (version 4.5.0) using the Bioconductor Annotation Dbi package (manipulation of SQLite-based annotations in Bioconductor, version 1.70.00), referencing org.Hs.eg.db (the genome-wide annotation for Humans, version 3.21.0), to annotate the gene list with their corresponding external gene name and Ensembl gene id. The external gene names were then queried in R using the Ensembl database version 101 and the Bioconductor package biomaRt (interface to BioMart databases, version 2.64.0) to further annotate the gene list with chromosome name, start position, end position and gene biotype [19].

### 2.4 ∣ Visualization

The data visualization plots generated in this study, including volcano plots, Venn diagrams and heatmaps, were produced in R (version 4.5.0) using the packages EnhancedVolcano (version 1.26.0), eulerr (version 7.0.2) and pheatmap (version 1.0.12), respectively.

### 2.5 ∣ Genomic Regions Enrichment of Annotations Tool (GREAT) Analysis

The GREAT analysis examines annotations of neighbouring genes to assign biological significance to noncoding genomic regions [14]. The GREAT database (version 4.0.4) was used for the analysis, using Human genomic coordinates (GRCh38), after providing the noncoding regions in BED format. The regions in the BED file were set as the test regions, with the whole genome as the background, using the application's standard settings. Statistical significance was assigned based on the Raw *p*-value, as the number of significant results was too few when based on FDR, leading to all results being interpreted as purely exploratory. Data were visualized as boxplots in R (version 4.5.0) using the ggplot2 package.

### 2.6 ∣ Receiver Operating Characteristic (ROC) Curve Analyses

Pairwise receiver operating characteristic (ROC) curve analyses were employed to assess the diagnostic performance of select lncRNAs as candidate biomarkers across all sample groups. In total, three pairwise comparisons were made, including MASLD versus Control, MASH versus Control and MASLD versus MASH. Youden's index was utilized to derive the area under the curve (AUC), 95% confidence interval (CI), sensitivity, specificity, accuracy and optimal threshold.

### 2.7 ∣ Weighted Gene Co-Expression Network Analysis (WGCNA)

Weighted gene co-expression network analysis (WGCNA) was employed to analyse co-expression patterns among lncRNAs and their associations with protein-coding genes. The analysis was performed using the WGCNA R package (Weighted Correlation Network Analysis, version 1.74) [20]. The GSE135251 count matrix was filtered to remove low-expression genes (minimum raw count < 10), before conducting normalization with the DESeq2 R package. The pickSoftThreshold() function and a signed co-expression network were employed to select the optimal soft threshold and identify highly co-expressed gene modules.

### 2.8 ∣ Database for Annotation, Visualization and Integrated Discovery (DAVID) Analysis

The bioinformatic tool Database for Annotation, Visualization and Integrated Discovery (DAVID) was utilized to conduct GO analysis of the genes included in all significant WGCNA modules [21]. The following categories were included in the analysis: GO: Biological Processes, GO: Molecular Function and GO: Cellular Components. The data were visualized in R using the ggplot2 and pheatmap packages for dot plots and heatmaps, respectively.

### 2.9 ∣ Independent Validation Cohort

A second validation dataset was obtained from NCBI-GEO under accession GSE126848, which comprises RNA-seq data from 57 liver biopsies. These samples included 14 healthy normal-weight controls and 12 obese individuals from the Center for Diabetes Research, Gentofte Hospital (University of Copenhagen, Hellerup, Denmark), as well as 15 MASLD patients and 16 MASH patients from the Department of Hepatology and Gastroenterology, Aarhus University Hospital (Aarhus, Denmark), as described in the publication by Suppli et al. [22]. The same end-to-end analytical pipeline used for our principal dataset was applied to this validation dataset. FASTQ files and series matrix files were downloaded from the NCBI GEO repository, and the DESeq2 R package was used to perform differential gene expression analysis. Gene annotations of the gene list obtained after the DESeq2 analysis were queried using the Bioconductor Annotation Dbi package to annotate the genes with their corresponding Ensembl gene id and external gene name, which were then queried in R using the Bioconductor package biomaRt to further annotate the genes with chromosome name, start position, end position and biotype. The gene list obtained from this validation dataset was then overlapped with the primary GSE135251 gene list to identify similar up- and down-regulated lncRNA signatures for both MASLD and MASH conditions.

## 3 ∣ Results

### 3.1 ∣ Observed Gene Expression Profiles in MASLD and MASH Patients

**FIGURE 1 ∣:**
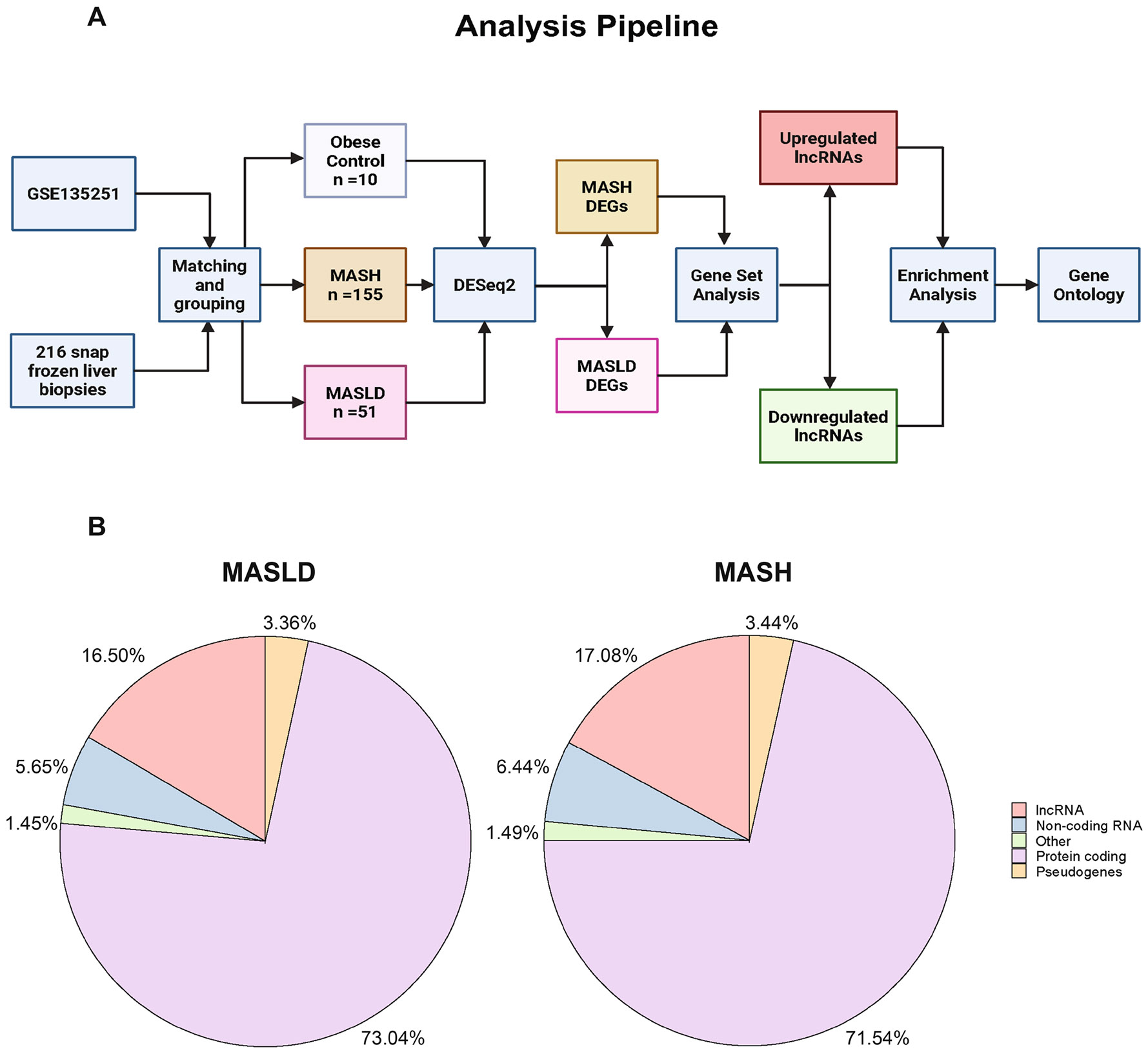
Study design and identified gene annotations. (A) Overview of the data analysis pipeline. (B) The percentage of biotypes that constitute the differentially expressed genes in MASLD and MASH.

A transcriptomic study by Govaere et al. [8] identified a 25-gene signature in diseased liver tissue associated with the progression of histologically defined MASLD and MASH. The study provided valuable insights into gene expression changes, particularly in protein-coding genes, that occur as the disease advances, which may serve as clinically relevant diagnostic and prognostic markers. In contrast, our analysis focused on identifying transcripts with limited protein-coding potential or noncoding RNAs that are differentially regulated in MASLD and MASH. To achieve this, we first extracted raw RNA-seq data from this study from the GEO database using the accession number GSE135251. We analysed it to identify all differentially expressed RNAs, including noncoding transcripts, in both study groups [8]. The raw read counts for MASLD and MASH groups were normalized to those of healthy obese control patients without evidence of MASLD (Figure 1A). After normalization, the resulting gene lists were annotated to distinguish and segregate genes by biotype (Figure 1B).

**FIGURE 2 ∣:**
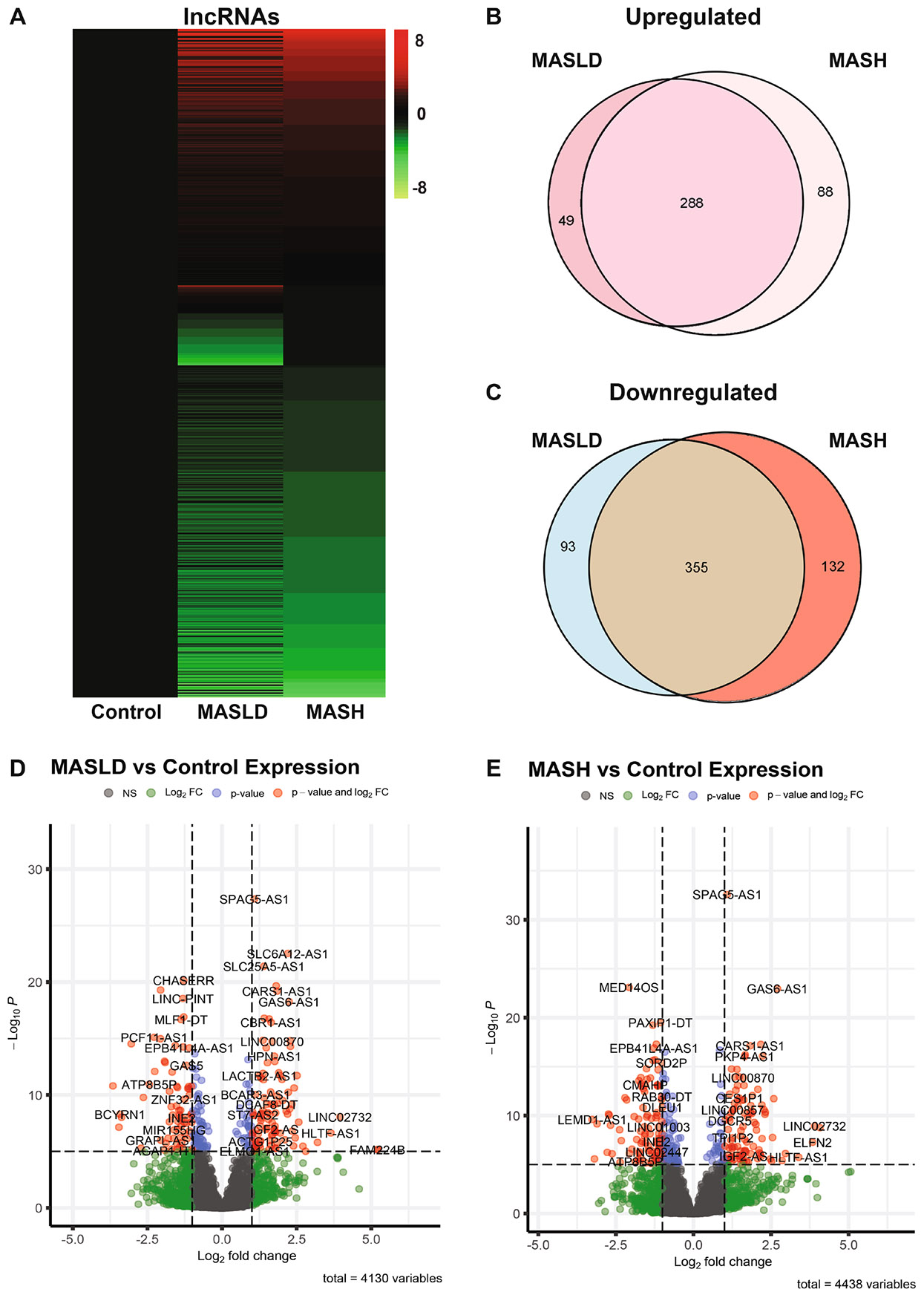
Visualization of differentially expressed noncoding genes in MASLD and MASH. (A) The RNA-seq expression patterns of differentially expressed genes (DEGs) in MASLD and MASH samples are illustrated in a heatmap on a log_2_(fold change) scale ranging from −8 to 8. The gradient scale indicates up-regulated (red) and down-regulated (green) regions in the dataset. Venn diagrams illustrate the overlaps of lncRNA DEGs in (B) up-regulated and (C) down-regulated genes across both sample types. Only genes with statistically significant differential expression (*p*-adjusted value < 0.05) are included in these analyses. Additionally, Volcano plots illustrate lncRNA expression in both (D) MASLD and (E) MASH samples. Genes with an absolute log_2_ fold change > 2 and a *p*-value ≤ 1 × 10^−6^ are represented in red.

To further focus on noncoding transcripts, such as long noncoding RNAs (lncRNAs), expressed during the transition from MASLD to MASH, we identified differentially expressed lncRNAs between the two groups. Differences in lncRNA gene regulation across the study cohorts are illustrated in a heatmap (Figure 2A). An intersectional Venn diagram of differentially expressed genes (DEGs) revealed that patients with MASLD exhibit up-regulation of 337 and down-regulation of 448 lncRNAs, respectively. In MASH patients, similar numbers were observed, with 376 up-regulated and 487 down-regulated lncRNAs. The overlaps included 288 and 355 lncRNAs that were up- and down-regulated across groups, respectively (Figure 2B,C, Table S1). Furthermore, analyses are more clearly visualized in volcano plots that display all statistically significant regulated lncRNA DEGs in patients with MASLD and MASH (Figure 2D,E, Table S1).

Tables 1 and 2 summarize the top 15 most prominently up- and down-regulated lncRNAs in each cohort. Despite some shared dysregulated lncRNA signatures, unique gene signatures persisted for each disease. Notably, among MASLD patients, the most prominent uniquely up-regulated lncRNA was *MYCNOS*. Conversely, the most highly down-regulated unique lncRNAs were *ST8SIA6-AS1* and *AADACL2-AS1* (Table 1). In MASH patients, the up-regulated lncRNAs included *ELFN2*, *LINC01435*, *LINC01507*, *GUCY1B2-206* and additional unlisted transcripts, while the most significant and distinct down-regulated lncRNA signatures were *SYBU-AS1*, *LGALS17A*, *ZNF474-AS1* and *LINC01116* (Table 2).

### 3.2 ∣ Assignment of Biological Meaning to Noncoding lncRNA Transcripts

**FIGURE 3 ∣:**
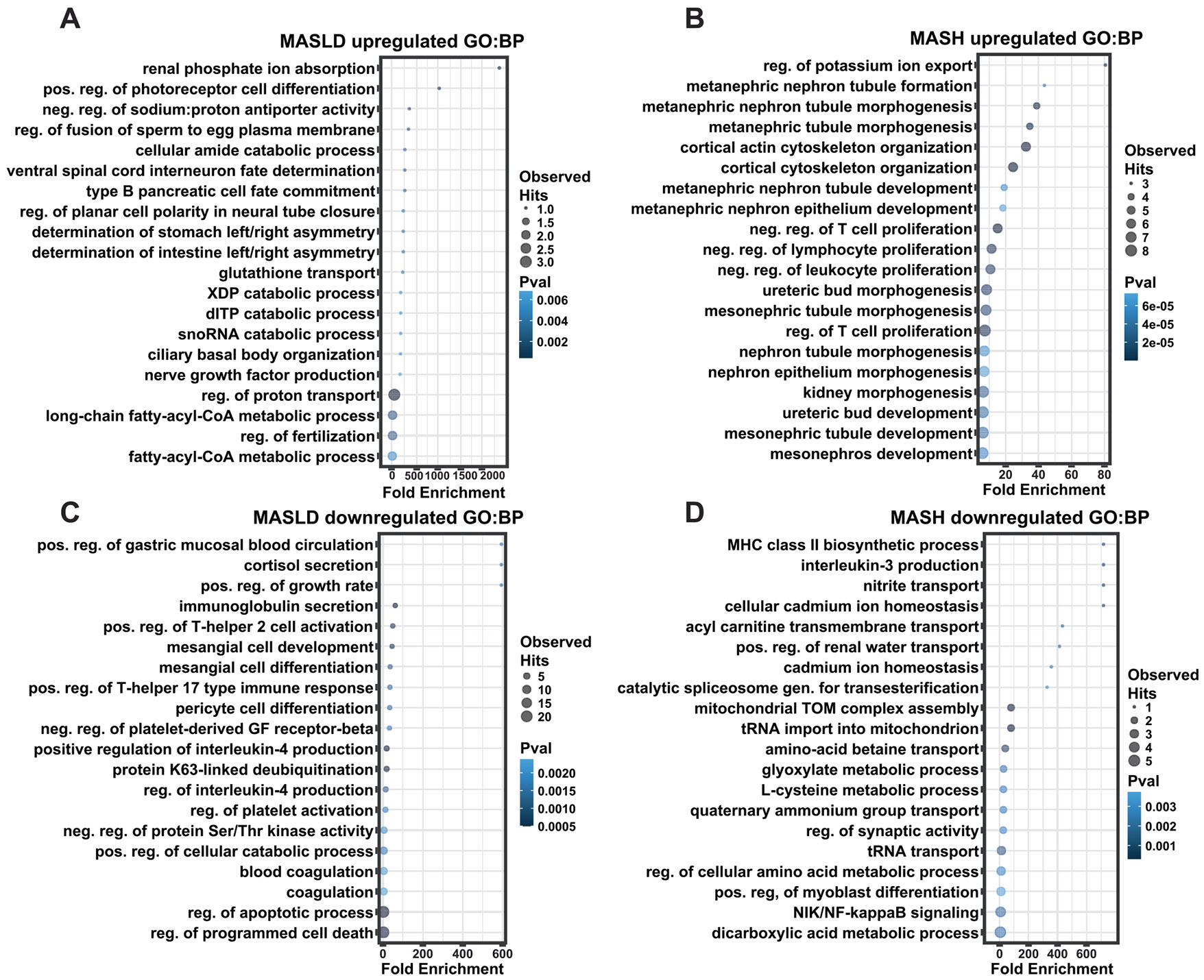
Analyses using GREAT predicts functions of cis-regulatory regions of noncoding genes for biological processes. (A) Genes up-regulated in MASLD; (B) genes up-regulated in MASH; (C) genes down-regulated in MASLD; (D) genes down-regulated in MASH.

**FIGURE 4 ∣:**
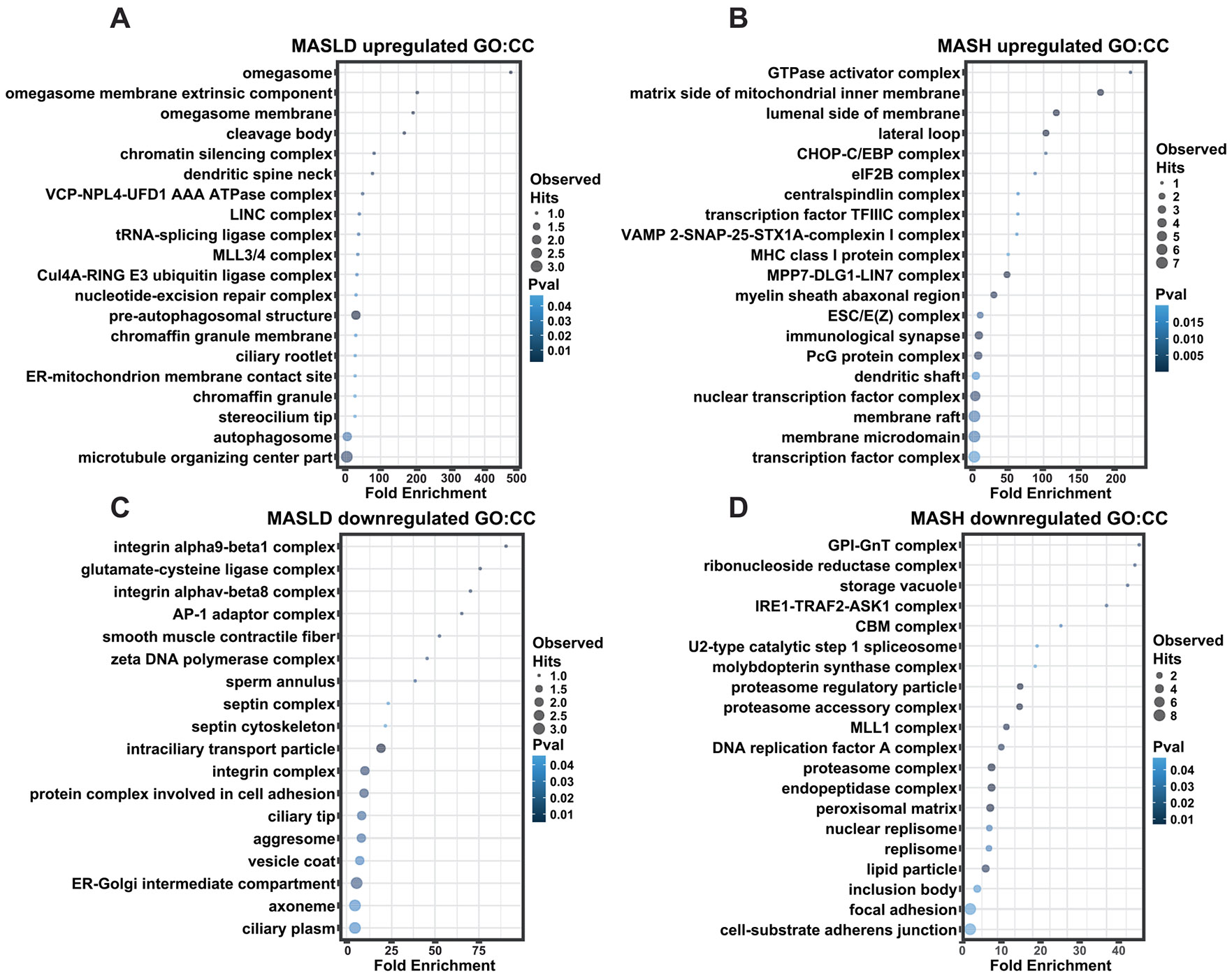
Analyses using GREAT predicts functions of cis-regulatory regions of noncoding genes for cellular components. (A) Genes up-regulated in MASLD; (B) genes up-regulated in MASH; (C) genes down-regulated in MASLD; (D) genes down-regulated in MASH.

**FIGURE 5 ∣:**
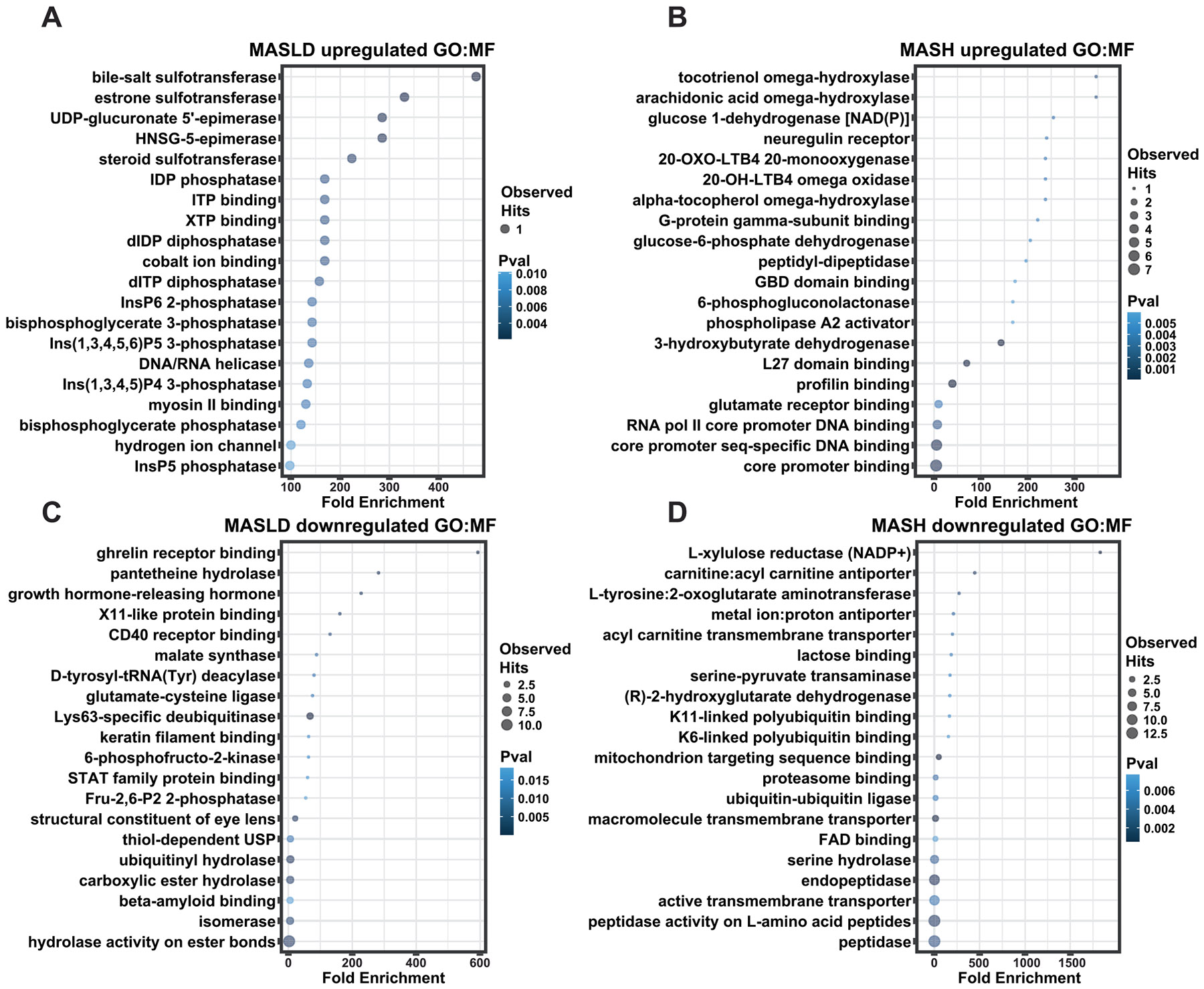
Analyses using GREAT predicts functions of cis-regulatory regions of noncoding genes for molecular functions. (A) Genes up-regulated in MASLD; (B) genes up-regulated in MASH; (C) genes down-regulated in MASLD; (D) genes down-regulated in MASH.

Given that the functions of lncRNA genes are less well characterized than those of protein-coding genes, a Genomic Regions Enrichment of Annotations Tool (GREAT) analysis was performed on the identified noncoding genes of interest. The GREAT analysis enriches genomic regions of interest and links them to putative biological functions [14]. After acquiring the genomic coordinates of the lncRNAs, these were uploaded to the GREAT web tool (http://great.stanford.edu). The analysis results revealed distinct enrichment patterns of noncoding genomic regions associated with the top up- and down-regulated lncRNA genes in both MASLD and MASH patients (Figures 3-5, Tables S2-S4).

In MASLD, the up-regulated lncRNAs were associated with biological processes (BPs) such as long-chain fatty-acyl-CoA and fatty-acyl-CoA metabolism, as well as regulation of fertilization and proton transport (Figure 3A). Conversely, the down-regulated genes were linked to the regulation of cellular catabolism, apoptosis and programmed cell death as well as blood coagulation (Figure 3C). In MASH, up-regulated lncRNAs were associated with kidney development, including mesonephros and ureteric bud formation, nephron morphogenesis and related processes (Figure 3B). Down-regulated genes in MASH were associated with dicarboxylic acid metabolism, amino acid metabolism, tRNA transport, myoblast differentiation and NIK/NF-kappa B signalling pathways (Figure 3D).

The GREAT analysis also predicted associations between lncRNAs and specific cellular components (CCs). Top CC terms for MASLD up-regulated lncRNAs included autophagosomes, microtubule-organizing centres, chromaffin granules and stereocilium tips (Figure 4A). Their down-regulated counterparts are linked to aggresomes, vesicle coats, ER-Golgi intermediate compartments, axonemes and cilia (Figure 4C). For MASH, up-regulated lncRNAs were associated with dendritic shafts, membrane rafts, lipid microdomains and transcription factor complexes (Figure 4B), while down-regulated lncRNAs were associated with replisomes, lipid particles, inclusion bodies, focal adhesions and adherens junctions (Figure 4D).

Finally, molecular function (MF) analysis indicated that up-regulated MASLD lncRNAs are associated with phosphatases such as Ins(1,3,4,5) P4 3-phosphatase, bisphosphoglycerate phosphatase and InsP5 phosphatase, as well as myosin II binding and proton channel activity (Figure 5A). The down-regulated lncRNAs were associated with ubiquitinyl and hydrolase activities, beta-amyloid binding and isomerase functions (Figure 5C). In MASH, the up-regulated lncRNAs were linked to profilin, glutamate and core-promoter binding, including RNA polymerase II core-promoter interactions (Figure 5B). In contrast, down-regulated lncRNAs were associated with peptidase and hydrolase activities, including serine and endopeptidases, as well as transmembrane transport functions (Figure 5D).

### 3.3 ∣ Assessment of the Diagnostic Performance of Select lncRNAs of Interest

**FIGURE 6 ∣:**
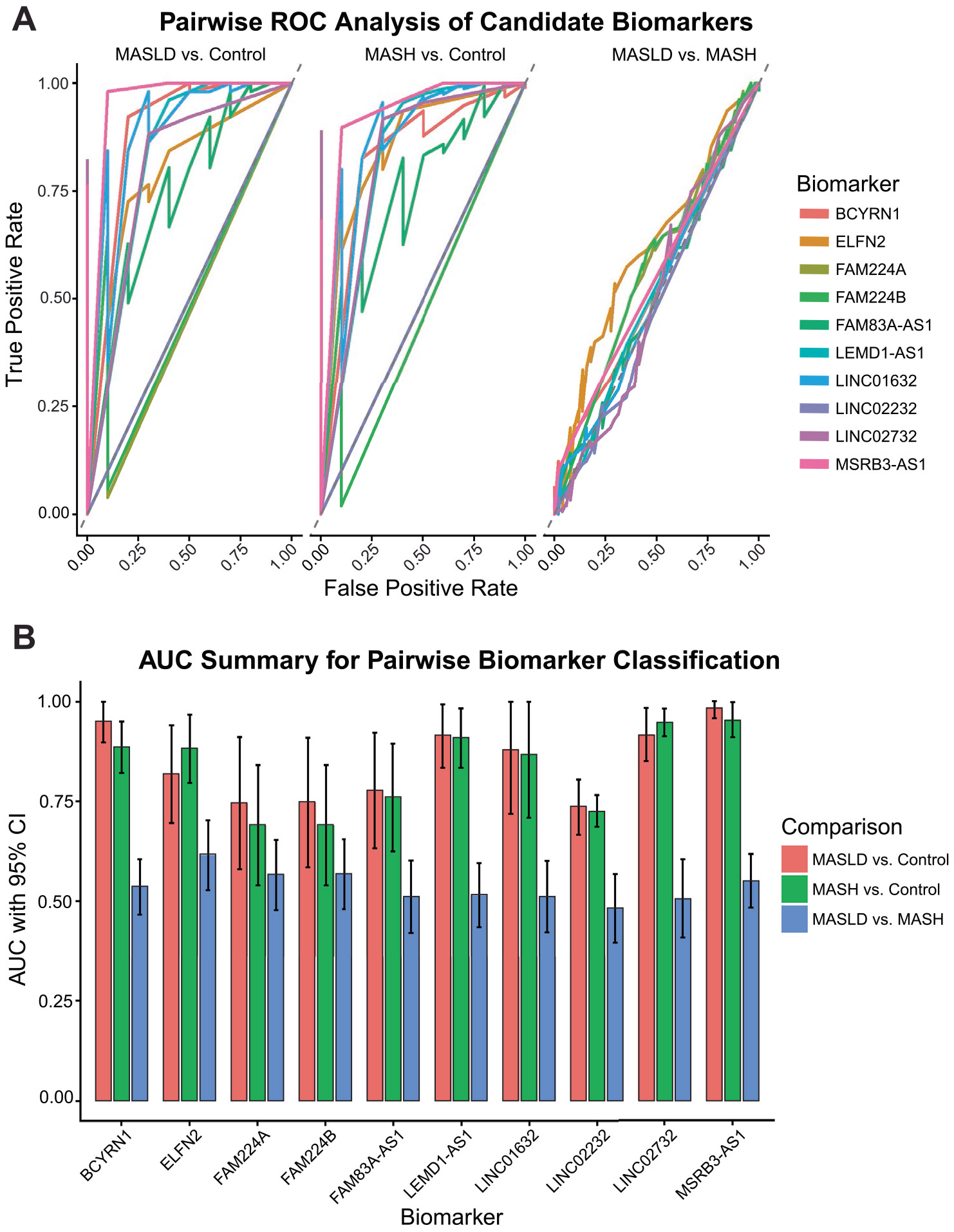
Comparison of ROC curve analyses to assess diagnostic performance of select lncRNAs. Pairwise ROC curve analyses of potential biomarkers were conducted across three relevant comparisons: MASLD versus Control, MASH versus Control and MASH versus MASLD. (A) The ROC curves and (B) AUC values were analysed for all 10 lncRNAs across the three comparison groups.

To assess the diagnostic performance of these lncRNAs as candidate biomarkers, pairwise receiver operating characteristic (ROC) curves and area under the curve (AUC) analyses were employed. While ROC analysis serves to assess the diagnostic performance of these biomarkers to diagnose the different conditions, the AUC value serves as a summary metric that reflects the ROCs ability to distinguish between individuals across condition states [23]. A total of three pairwise comparisons were made, including MASLD versus Control, MASH versus Control and MASLD versus MASH (Figure 6A). All the biomarkers evaluated showed high diagnostic value for distinguishing MASLD versus Control and MASH versus Control (AUC = 0.85–0.95), but limited discriminatory ability between MASLD and MASH (AUC = 0.50–0.60) (Figure 6B). These results suggest that while the biomarkers are good indicators of liver disease, they may have limited capacity to distinguish disease progression.

Among the differentially expressed lncRNAs in MASLD and MASH, the top five up-regulated genes in both conditions included four shared lncRNAs (*FAM224A*, *FAM224B*, *LINC02232* and *LINC02732*). Notably, among the top 10 up-regulated lncRNAs in MASH, *ELFN2-208* was up-regulated in a statistically significant manner in this condition and not in MASLD. Similarly, among the top five down-regulated categories, three lncRNAs were common in both conditions: *MSRB3-AS1*, *LINC01632* and *LEMD1-AS*. Among the top five, *BCYRN1* was down-regulated in both conditions, though its log_2_FC value was double in the MASLD condition compared to MASH (Figure S1). When the top 10 down-regulated lncRNAs are considered, *FAM83A-AS1* also demonstrated down-regulation in both conditions, though its log_2_FC value was double in the MASH condition compared to MASLD (Figure S1).

### 3.4 ∣ Co-Expression Analysis and Independent Cohort Validation

Because the functional inferences from our GREAT analysis are inherently predictive, we conducted additional analyses to strengthen the validity of our findings. A Weighted Gene Co-expression Network Analysis (WGCNA) was employed to construct weighted correlation networks between lncRNAs and protein-coding genes across MASLD and MASH samples (Figure S2A). Of all the co-expression modules generated by the analysis, only the black module was selected for functional annotation because it reached statistical significance across Control, MASLD and MASH samples (Figure S3). Thus, all genes included in this module were selected for a secondary Gene Ontology (GO) analysis and queried utilizing the Database for Annotation, Visualization and Integrated Discovery (DAVID) bioinformatic tool. The Gene Ontology terms from DAVID were overlaid with the previously discussed GREAT terms to identify common terms that contribute to the validity of our interpretations, which are displayed in a series of intersectional Venn Diagrams and dot plots (Figures S2B and S4-S6). The list of terms overlapping in Figure S2B can be shown in Table S5.

To further enhance confidence in the mechanistic interpretation of our GO findings, we analysed a second liver transcriptomic study by Suppli et al. [22] to serve as an independent validation cohort. The same end-to-end analysis pipeline was applied to this dataset, with all appropriate raw RNA-seq data files extracted from the GEO database using accession number GSE126848. Raw read counts from the MASLD and MASH groups were normalized to healthy patients, and the resulting gene lists were annotated to identify all differentially expressed lncRNAs in each sample group. The resulting gene lists were overlaid with the up- and down-regulated lncRNA lists from the GSE135251 dataset to identify lncRNAs differentially expressed in both datasets. Intersectional Venn diagrams revealed 24 common up-regulated lncRNAs in the MASLD condition and 69 up-regulated in the MASH condition (Figures S7A and S9A). In contrast, 34 common down-regulated lncRNAs were observed in the MASLD condition, and 25 down-regulated in the MASH condition (Figures S8A and S10A). These common lncRNA lists for each condition were subjected to GREAT analysis to predict their putative biological functions (Figures S7-S10).

## 4 ∣ Discussion

While prior studies have implicated lncRNAs in MASLD, most have focused primarily on experimental models to explore their role, with limited biopsy-based analyses [24, 25]. Our study expands on clinical studies by profiling lncRNA expression in a large set of patient samples from MASLD and MASH patients, identifying transcripts that are consistently dysregulated in early versus advanced disease.

Among the aberrantly expressed lncRNAs across both conditions, four are shared (*FAM224A*, *FAM224B*, *LINC02232* and *LINC02732*). This overlap suggests the potential existence of common regulatory mechanisms in the progression from MASLD to MASH. However, the functional roles of these lncRNAs in liver disease remain largely unexplored. For instance, *FAM224A* has been studied in glioblastoma [26], but its function in metabolic or hepatic pathways remains unknown.

Interestingly, *ELFN2-208* was up-regulated in a statistically significant way in the MASH condition and not in MASLD. The *ELFN2-208* lncRNA is associated with the extracellular leucine-rich repeat and fibronectin type III domain-containing 2 (ELFN2) gene [27]. This gene has been reported to alter synaptic structure and function, and to affect neurotransmission [27]. In cancer, the *ELFN2* gene has been reported to promote cancer survival and metastasis in certain tumours [28]. In our study, *ELFN2-208* was up-regulated explicitly in MASH samples, but not in MASLD, suggesting that it may reflect disease-stage-specific regulation. Interestingly, given that patients with advanced liver disease and cirrhosis often experience elevated ammonia levels and altered brain function [29], the up-regulation of a transcript regulating a synaptic protein raises the possibility that *ELFN2-208* could be linked to neurological changes observed in advanced MASLD. While speculative, future studies are needed to determine whether *ELFN2-208* contributes mechanistically to disease progression or reflects the activation of pathways that could be therapeutically targeted to prevent MASLD from advancing to MASH.

Similarly, among the top five down-regulated categories, three lncRNAs were shared between the two conditions. These lncRNAs, similar to the overlapping lncRNAs in the up-regulated list, suggest common regulatory mechanisms in disease progression. Among the top five, *BCYRN1* was down-regulated twice as much in MASLD compared to the MASH condition, which is something that seems to align with other previous studies. *BCYRN1* has been previously linked to inflammation [30], and its up-regulation has been associated with HCC [31], highlighting the potential importance of its reduced expression in early disease. In our analyses, *FAM83A-AS1* was down-regulated in both MASLD and MASH samples. However, He and Yu [32] previously demonstrated in HCC that *FAM83A-AS1* is up-regulated and functions as an oncogene, promoting cell proliferation, migration and survival by stabilizing its target gene, *FAM83A*, through NOP58 binding. This discrepancy may reflect stage-specific or context-dependent roles for this lncRNA, where its expression is suppressed during metabolic liver disease but later up-regulated during malignant transformation. Alternatively, this pattern may suggest that activation of *FAM83A-AS1* is associated with progression towards HCC rather than earlier disease stages. However, the biological significance of this opposing expression pattern remains unclear, and these observations should be considered hypothesis-generating. Further experimental studies will be required to determine whether *FAM83A-AS1* plays distinct roles across disease progression or contributes to the transition from MASLD/MASH to HCC.

As shown, multiple lncRNAs are differentially expressed in MASLD and MASH, many of which remain poorly characterized in the existing literature. Therefore, we conducted a GREAT analysis to examine the biological processes associated with the differentially expressed lncRNAs to gain insights into the potential mechanisms driving MASLD progression to MASH. Up-regulated lncRNAs in MASLD were plausibly associated with biological processes related to long-chain fatty-acyl-CoA and fatty-acyl-CoA metabolism, suggesting increased lipid processing and accumulation in the liver, consistent with the established role of dysregulated lipid metabolism as a hallmark of this condition [33, 34]. In parallel, down-regulated lncRNAs were linked to cellular catabolism, which is associated with programmed cell death [35]. These findings appear to elucidate a coordinated shift in hepatic metabolic regulation, where enhanced lipid synthesis and fatty-acyl-CoA metabolism may synergize with the suppression of catabolic pathways, potentially contributing to lipid accumulation and disease progression. This mechanistic interplay aligns with established features of MASLD and MASH, where disrupted lipid metabolism leads to fat accumulation, inflammation and liver scarring [36].

In addition to biological processes, up-regulated lncRNAs in MASLD were associated with specific cellular components, including autophagosomes, suggesting a potential role in regulating autophagy, which is critical for lipid turnover and cellular homeostasis in the liver [37]. In a study by Ding et al., hepatic autophagy was reported to be enhanced in early stages of MASLD but blocked in later stages, both in vivo and in vitro [38], supporting the idea that dysregulation of autophagic pathways contributes to disease progression and hepatocyte stress. Our analysis also showed that the microtubule-organizing centres (MTOCs), which regulate cytoskeletal dynamics and microtubule stability, were associated with up-regulated lncRNAs. Previous studies have shown that microtubule destabilization promotes cytoskeletal rearrangement and hepatic stellate cell activation during fibrosis [39]. Similarly, ethanol-induced microtubule acetylation alters lipid droplet functions, contributing to enhanced hepatic steatosis [40]. These studies support our findings that lncRNAs enriched for MTOCs could influence cytoskeletal remodelling and lipid functions, contributing to both fibrotic and metabolic changes in MASLD.

Additionally, chromaffin granules, a cellular component typically located in the adrenal medulla, have been identified adjacent to the liver in other species and are associated with lncRNAs up-regulated in MASLD [41]. These granules are associated with glucocorticoid secretion and dysregulated glucocorticoid signalling, which have been linked to MASLD development and hepatic lipid accumulation [42]. These findings suggest that the up-regulated lncRNAs associated with the chromaffin granules may influence glucocorticoid-mediated metabolic regulation in MASLD. Another cellular component was the stereocilium tip, a structure critical to mechanosensory function in the inner ear [43]. There have been studies linking metabolic dysfunction and hearing loss, proposing a ‘liver-ear axis’ relation likely through impaired inner-ear vascular structures, systemic inflammation and oxidative stress [44].

There were also cellular components associated with down-regulated lncRNAs in MASLD. One such component was the aggresome, which assists in the degradation of misfolded or aggregated proteins [45]. They are formed near MTOCs and are transported along the microtubule network. Interestingly, in our study, MTOCs were associated with up-regulated lncRNAs in the MASLD condition, whereas aggresome-associated lncRNAs were down-regulated, suggesting dysregulation of the coordination between microtubule organization and protein processing. This imbalance may contribute to hepatocyte stress and disease progression. Another MASLD-linked component associated with down-regulated lncRNAs was the vesicle coat, a key component of vesicular trafficking and secretion. Extracellular vesicles have been investigated and associated with MASLD pathogenesis [46]. More specifically, coat protein complex II (COPII) has been shown to play a crucial role in maintaining hepatic metabolic homeostasis. Its disruption activates the unfolded protein response and drives hepatic steatosis [47]. Thus, the down-regulation of vesicle-coat-associated lncRNAs may reflect impaired intracellular trafficking that could exacerbate metabolic stress and contribute to disease progression. Additional down-regulated cellular components included: the ER-Golgi intermediate compartments, axonemes and cilia. These organelles are part of intracellular trafficking and cellular communication. The ER-Golgi intermediate compartment has been associated with ER stress and altered metabolic homeostasis in liver disease [48]. Similarly, axonemes and cilia are critical for sensing extracellular signals and coordinating pathways involved in lipid metabolism and inflammation [49]. Dysregulation of hepatic cilia has been linked to obesity and shown to contribute to MASLD progression by impairing insulin signalling [50]. While cilia-related components were linked to down-regulated lncRNAs, we observed enrichment of terms such as stereocilium tips among up-regulated lncRNAs. However, it is important to interpret these findings with caution, as GREAT-based functional annotations rely on genomic proximity and may reflect associations with developmental genes that are not actively expressed in the adult liver.

Although epidemiological studies have reported an increased incidence of hearing impairment in individuals with metabolic liver disease, suggesting a potential systemic link, the biological relevance of stereocilium-related annotations in this context remains uncertain [44, 51]. Therefore, these observations should be considered hypothesis-generating, and further functional studies will be required to determine whether these associations reflect meaningful regulatory mechanisms or are an artefact of genomic proximity.

Similarly, we observed many cellular components associated with up-regulated lncRNAs in the MASH condition. One of the cellular components was the dendritic shaft, which represents an extension of dendritic cells [52]. Hepatic dendritic cells play a central role in maintaining immune tolerance and hepatic homeostasis by secreting anti-inflammatory cytokines and promoting hepatic stellate cell quiescence [53]. Given this information, the up-regulation of lncRNAs associated with dendritic shafts may reflect an immune-driven shift that underlies the chronic inflammation characteristic of MASH. Membrane shafts and lipid microdomains were also up-regulated, potentially signalling an alteration in membrane organization that favours lipid accumulation and inflammatory signalling [54, 55]. The association of transcription factor complexes with up-regulated lncRNAs suggests their roles in transcriptional regulation in hepatocytes and in the control of inflammatory pathways [56]. On the other hand, cellular components such as replisomes, which are responsible for DNA replication and maintaining cellular integrity, were associated with down-regulated lncRNAs [57]. Dysregulation in replisome function can disrupt DNA replication and affect hepatocyte viability. Mitochondria have their own DNA (mtDNA), which is replicated by a specialized mitochondrial replisome; dysregulation of this process has been linked to the pathogenesis of liver disease [58]. Thus, the observed replisome-associated down-regulated lncRNAs may be due to compromised mitochondrial function, potentially exacerbating hepatocellular stress and facilitating disease progression. Also, lipid particles were associated with down-regulated lncRNAs in MASH, and alterations in lipid particles have been studied and linked to the progression of fatty liver disease [59]. In our analysis, inclusion bodies were related to down-regulated lncRNAs, which was unexpected given their established role in liver pathology. Mallory-Denk bodies (MDBs) are cytoplasmic inclusions and have been considered a hallmark of MASH and fibrosis progression, aiding in the diagnosis of liver tissue [60]. This apparent discrepancy may reflect context-dependent regulation, in which a different form of inclusion body formation may be involved in MASH progression, and further studies may help elucidate this unexpected finding. The cellular component, focal adhesions, was associated with down-regulated lncRNAs in our analysis. The role of focal adhesions in liver disease is complex, as they can be either up-regulated or down-regulated depending on the cellular context. For instance, in a study by Feng et al., Kindlin-2, Filamin A and Paxillin have been shown to promote fibrosis progression. In contrast, deletion of focal adhesion kinase (FAK) in liver cells increased fibrosis, contrary to what might be expected [61]. These findings suggest that the differential association of cellular components with down-regulated lncRNAs presented in our analysis is context-dependent. Another cellular component, adherens junctions, was associated with down-regulated lncRNAs in MASH. They are essential to maintaining tissue integrity by linking neighbouring cells through cadherin-catenin complexes and connecting to actin filaments [62]. Their association with differentially expressed lncRNAs suggests that lncRNAs might contribute to altered hepatocyte organization and impaired liver architecture during disease progression.

A major limitation of this study is the lack of experimental validation of the identified lncRNA candidates. As this work was designed as a computational, hypothesis-generating analysis, validation in independent patient cohorts and in vitro models will be essential to confirm the reproducibility and functional relevance of these findings. However, to substantiate the biomarker potential of the candidate lncRNAs identified in the study, we performed an ROC curve analysis to quantitatively evaluate their diagnostic performance across three clinically relevant comparisons. We observed strong discriminatory ability when comparing disease groups to healthy controls, suggesting that these transcripts may reflect meaningful transcriptional changes associated with the onset and progression of metabolic liver disease. However, the limited capacity of these biomarkers to distinguish MASLD from MASH underscores a well-recognized challenge: molecular signatures of disease presence do not necessarily translate into markers of disease stage. These findings suggest that combinatorial biomarker panel approaches, rather than individual transcript evaluation, will likely be necessary to achieve the sensitivity and specificity required for accurate clinical staging of metabolic liver disease progression.

Complementary co-expression network analysis using WGCNA was also performed, and it further reinforced our findings, identifying biologically coherent modules linking candidate lncRNAs to protein-coding genes involved in Wnt signaling, immune regulation and wound response pathways. The convergence of GREAT and WGCNA-derived annotations provides stronger mechanistic grounding for the functional relevance of the lncRNA candidates identified in this study.

Validation is essential to determine how these lncRNAs mechanistically affect the progression of MASLD to MASH and to assess their potential as therapeutic targets. Advances in RNA therapeutics, such as antisense oligonucleotides (ASOs), have made it increasingly feasible to interrogate lncRNA function [63]. In addition, 3D liver organoid models can help mimic the in vivo hepatic environment and assess how modulation of these lncRNAs affects disease pathways [64]. While the present study is computational in nature, the concordance observed between two independent RNA-seq cohorts supports the robustness of the identified transcriptional signatures. In our future work, we aim to validate key lncRNAs in vitro and in vivo and explore their potential to advance translational approaches for liver metabolic diseases.

In summary, our study highlights distinct patterns of lncRNA dysregulation across MASLD and MASH, spanning biological processes, cellular components, molecular functions and metabolic pathways. These results underscore the complexity of lncRNA-mediated regulation in liver disease. This work provides a foundation for future mechanistic studies and therapeutic exploration.

## Supplementary Material

Figures S1-S10

Table S1

Table S2

Table S3

Table S4

Table S5

Additional supporting information can be found online in the Supporting Information section. **Figure S1:** Identification of a 23-lncRNA gene set signature. The expression patterns of statistically significant lncRNAs (*p*adj < 0.05) that demonstrated a 0.5 or more log_2_(-fold change) difference between the MASLD and MASH conditions are illustrated using a heatmap. A log_2_(fold change) scale ranging from 4 to −4 is included, which indicates the up-regulated (red) and down-regulated (green) regions in the dataset. **Figure S2:** WGCNA of protein and noncoding genes provides reinforcement of GREAT GO terms. (A) Hierarchical cluster tree showing the 12 modules of co-expressed genes identified in the GSE135251 dataset. The lower panel of the graph depicts all modules, which are marked by specific colours, such as ‘MEturquoise’ and ‘MEblue’. Co-expressed genes in statistically significant modules were submitted to DAVID to obtain GO terms. DAVID GO terms were compared to GREAT terms for all DEG lncRNAs in both sample types. Intersection Venn diagrams illustrate the DAVID and GREAT term overlaps in (B) MASLD and (C) MASH. **Figure S3:** WGCNA Module-trait relationship analysis of protein and noncoding genes. Heatmap represents the module-trait relationship of the WGCNA analysis. The colour scale on the right shows module-trait correlation from −1 (blue) to 1 (red), with corresponding *p*-values included in parentheses. **Figure S4:** Biological Processes Gene Ontology overlapping terms from DAVID and GREAT analyses. Co-expressed genes in statistically significant modules were submitted to DAVID to obtain Biological Processes GO terms. These terms were compared to GREAT Biological Processes terms for (A) lncRNAs up-regulated in MASLD; (B) lncRNAs up-regulated in MASH; (C) lncRNAs down-regulated in MASLD; (D) lncRNAs down-regulated in MASH, to identify overlapping terms. **Figure S5:** Cellular Component Gene Ontology overlapping terms from DAVID and GREAT analyses. Co-expressed genes in statistically significant modules were submitted to DAVID to obtain Cellular Component GO terms. These terms were compared to GREAT Cellular Component terms for (A) lncRNAs up-regulated in MASLD; (B) lncRNAs up-regulated in MASH; (C) lncRNAs down-regulated in MASLD; (D) lncRNAs down-regulated in MASH, to identify overlapping terms. **Figure S6:** Molecular Function Gene Ontology overlapping terms from DAVID and GREAT analyses. Co-expressed genes in statistically significant modules were submitted to DAVID to obtain Molecular Function GO terms. These terms were compared to GREAT Molecular Function terms for (A) lncRNAs up-regulated in MASLD; (B) lncRNAs up-regulated in MASH; (C) lncRNAs down-regulated in MASLD; (D) lncRNAs down-regulated in MASH, to identify overlapping terms. **Figure S7:** Identification and function prediction of up-regulated MASLD lncRNAs across two datasets. Up-regulated lncRNA gene lists in MASLD samples were obtained from both the principal (GSE135251) and validation dataset (GSE126848). (A) Intersection Venn diagrams illustrate the lncRNA overlaps between both datasets. GREAT analysis was employed to predict the functions of these overlapping genes for (B) biological processes, (C) cellular components and (D) molecular functions. **Figure S8:** Identification and function prediction of down-regulated MASLD lncRNAs across two datasets. Down-regulated lncRNA gene lists in MASLD samples were obtained from both the principal (GSE135251) and validation dataset (GSE126848). (A) Intersection Venn diagrams illustrate the lncRNA overlaps between both datasets GREAT analysis was employed to predict the functions of these overlapping genes for (B) biological processes, (C) cellular components and (D) molecular functions. **Figure S9:** Identification and function prediction of up-regulated MASH lncRNAs across two datasets. Up-regulated lncRNA gene lists in MASH samples were obtained from both the principal (GSE135251) and validation dataset (GSE126848). (A) Intersection Venn diagrams illustrate the lncRNA overlaps between both datasets. GREAT analysis was employed to predict the functions of these overlapping genes for (B) biological processes, (C) cellular components and (D) molecular functions. **Figure S10:** Identification and function prediction of down-regulated MASH lncRNAs across two datasets. Down-regulated lncRNA gene lists in MASLD samples were obtained from both the principal (GSE135251) and validation dataset (GSE126848). (A) Intersection Venn diagrams illustrate the lncRNA overlaps between both datasets GREAT analysis was employed to predict the functions of these overlapping genes for (B) biological processes, (C) cellular components and (D) molecular functions. **Table S1:** Information for up- and down-regulated lncRNA gene lists visualized in a Venn diagram. Lists of the ENSEMBL gene IDs and log_2_ fold-change values for all lncRNAs in each category of the Figure 2 up- and down-regulated Venn Diagrams. The categories, which consist of all the lncRNAs included in the figure, those common to both conditions, and those unique to the MASLD or MASH conditions, are included as separate sheets of the file. **Table S2:** Information on the GREAT-predicted functions of noncoding genes for biological processes. Data points for all the GREAT biological process categories of the lncRNA genes up- and down-regulated in MASLD and MASH. **Table S3:** Information on the GREAT-predicted functions of noncoding genes for cellular components. Data points for all the GREAT cellular component categories of the lncRNA genes up- and down-regulated in MASLD and MASH. **Table S4:** Information on the GREAT-predicted functions of noncoding genes for molecular functions. Data points for all the GREAT biological process categories of the lncRNA genes up- and down-regulated in MASLD and MASH. **Table S5:** Information for the overlapping GREAT and DAVID terms of noncoding genes. GREAT Data points for all the biological processes, cellular components and molecular functions terms that overlapped with DAVID GO terms for the MASLD and MASH conditions.

## Figures and Tables

**TABLE 1 ∣:** List of top up- and down-regulated lncRNAs in MASLD samples relative to healthy controls.

No.	Up-regulated genes			Down-regulated genes		
	ENSEMBL ID	Gene symbol	log_2_(fold change)	ENSEMBL ID	Gene symbol	log_2_(fold change)
1	ENSG00000233522	*FAM224A*	5.218478889	ENSG00000250748	*MSRB3-AS1*	−3.657252678
2	ENSG00000230663	*FAM224B*	5.206125597	ENSG00000277199	*LINC01632*	−3.456487488
3	ENSG00000250125	*LINC02232*	4.061359666	ENSG00000236824	*BCYRN1*	−3.421170061
4	ENSG00000254416	*LINC02732*	3.954503118	ENSG00000226235	*LEMD1-AS1*	−3.369514166
5	ENSG00000235412	*TTTY4B*	3.883199108	ENSG00000224857	*LINC01691*	−3.042819873
6	ENSG00000228296	*TTTY4C*	3.876312144	ENSG00000236054	*SYN3-AS1*	−2.958063466
7	ENSG00000226906	*TTTY4*	3.848641735	ENSG00000233208	*LINC00642*	−2.713745891
8	ENSG00000239718	*HLTF-AS1*	3.645975794	ENSG00000245534	*RORA-AS1*	−2.635840066
9	ENSG00000255774	*LINC02747*	3.198501716	ENSG00000179428	*IL6-AS1*	−2.621072783
10	ENSG00000229236	*TTTY10*	3.188474458	ENSG00000242908	*AADACL2-AS1*	−2.602566031
11	ENSG00000256151	*ADGRD1-AS1*	2.798982148	ENSG00000257345	*LINC02413*	−2.521397865
12	ENSG00000250358	*LINC02200*	2.764791409	ENSG00000204832	*ST8SIA6-AS1*	−2.483603288
13	ENSG00000233718	*MYCNOS*	2.742820622	ENSG00000290567	*ATP8B5P-202*	−2.438460143
14	ENSG00000224821	*COL4A2-AS2*	2.712180443	ENSG00000235997	*LINC01936*	−2.43104561
15	ENSG00000249906	*NGFR-AS1*	2.708346489	ENSG00000197595	*ATP11AUN*	−2.426217451

*Note:* LncRNA genes in red or green colour were up-regulated and down-regulated in MASLD patients, respectively. These signatures are unique to this condition and were not observed in the top dysregulated MASH gene signatures.

**TABLE 2 ∣:** List of top up- and down-regulated lncRNA genes in MASH samples relative to healthy controls.

No.	Up-regulated genes			Down-regulated genes		
	ENSEMBL ID	Gene symbol	log_2_(fold change)	ENSEMBL ID	Gene symbol	log_2_(fold change)
1	ENSG00000233522	*FAM224A*	5.068285935	ENSG00000226235	*LEMD1-AS1*	−3.265640567
2	ENSG00000230663	*FAM224B*	5.001985393	ENSG00000277199	*LINC01632*	−3.190761411
3	ENSG00000255774	*LINC02747*	4.077373111	ENSG00000250748	*MSRB3-AS1*	−3.108300428
4	ENSG00000250125	*LINC02232*	3.948376558	ENSG00000236054	*SYN3-AS1*	−2.779884929
5	ENSG00000254416	*LINC02732*	3.903308773	ENSG00000204949	*FAM83A-AS1*	−2.73935221
6	ENSG00000243902	*ELFN2-208*	3.838986859	ENSG00000262466	*AADACL2-AS1*	−2.717644063
7	ENSG00000235412	*TTTY4B*	3.691259476	ENSG00000224857	*LINC01691*	−2.67467553
8	ENSG00000228296	*TTTY4C*	3.670419085	ENSG00000248050	*SYBU-AS1*	−2.622684257
9	ENSG00000226906	*TTTY4*	3.665435106	ENSG00000291086	*LGALS17A-207*	−2.608967166
10	ENSG00000239718	*HLTF-AS1*	3.392539383	ENSG00000257345	*LINC02413*	−2.574551189
11	ENSG00000250358	*LINC02200*	3.187745028	ENSG00000179428	*IL6-AS1*	−2.496111733
12	ENSG00000230945	*LINC01507*	3.18540444	ENSG00000249610	*ZNF474-AS1*	−2.45155258
13	ENSG00000229981	*LINC01435*	3.148401031	ENSG00000163364	*LINC01116*	−2.434828332
14	ENSG00000293412	*GUCY1B2-206*	3.011884683	ENSG00000266256	*LINC00683*	−2.429319284
15	ENSG00000229236	*TTTY10*	2.978250026	ENSG00000254139	*LINC03018*	−2.382551037

*Note:* LncRNA genes in red or green colour were up-regulated and down-regulated in MASH patients, respectively. These signatures are unique to this condition and were not observed in the top dysregulated MASLD gene signatures.

## Data Availability

The dataset for this study's analysis was obtained from NCBI's Gene Expression Omnibus (GEO) database (https://www.ncbi.nlm.nih.gov/geo/) on June 24, 2025, with the superseries accession number GSE135251, and on May 01, 2026, with the superseries accession number GSE126848.

## Keypoints

- Long noncoding RNAs (lncRNAs) remain largely unexplored in the progression of metabolic dysfunction-associated steatotic liver disease (MASLD) to hepatocellular carcinoma (HCC).
- Transcriptomic analysis of publicly available datasets revealed distinct lncRNA dysregulation profiles in MASLD and MASH, with both condition-specific and shared expression signatures.
- Unique lncRNA signatures were identified for both conditions, and GREAT analyses were applied to infer the biological relevance of these genes.
- ROC and AUC analyses indicate that a subset of lncRNAs can be used as diagnostic biomarkers.
