# Supplementary material for: Noncoding RNA Transcripts Based Molecular Phenotyping of MASLD and MASH Patients
Source: Liver Int Commun. Author manuscript; Available in PMC 2026 Aug 9. (PMC13451033; doi:10.1002/lci2.70053)
Supplement: Supplementary file 1 [file NIHMS2196815-supplement-Figures_S1-S10.pdf]

Supplementary Information

"Noncoding RNA Transcripts Based Molecular Phenotyping of MASLD and MASH Patients "

***Delgadillo et al.* (2026)**

This document contains the following supplementary information: Page

Supplementary Figures 2

•Suppl. Figure 1. Identification of a 23-lncRNA gene set signature…………………………..2
•Suppl. Figure 2. WGCNA of protein and non-coding genes provides reinforcement of GREAT GO terms……………………………………………………………………………….3
•Suppl. Figure 3. WGCNA Module-trait relationship analysis of protein and non-coding genes.…………………………………………………………………………….……….4
•Suppl. Figure 4. Biological Processes Gene Ontology overlapping terms from DAVID and GREAT analyses ………………………………………………………………………………5
•Suppl. Figure 5. Cellular Component Gene Ontology overlapping terms from DAVID and GREAT analyses ………………………………………………………………………………6
•Suppl. Figure 6. Molecular Function Gene Ontology overlapping terms from DAVID and GREAT analyses ………………………………………………………………………………7
•Suppl. Figure 7. Identification and function prediction of upregulated MASLD lncRNAs across two datasets..……………………………………………………………………….8
•Suppl. Figure 8. Identification and function prediction of downregulated MASLD lncRNAs across two datasets..……………………………………………………………………….9
•Suppl. Figure 9. Identification and function prediction of upregulated MASH lncRNAs across two datasets..………………………………………………………………………10
•Suppl. Figure 10. Identification and function prediction of downregulated MASH lncRNAs across two datasets……………………………………………………….................11

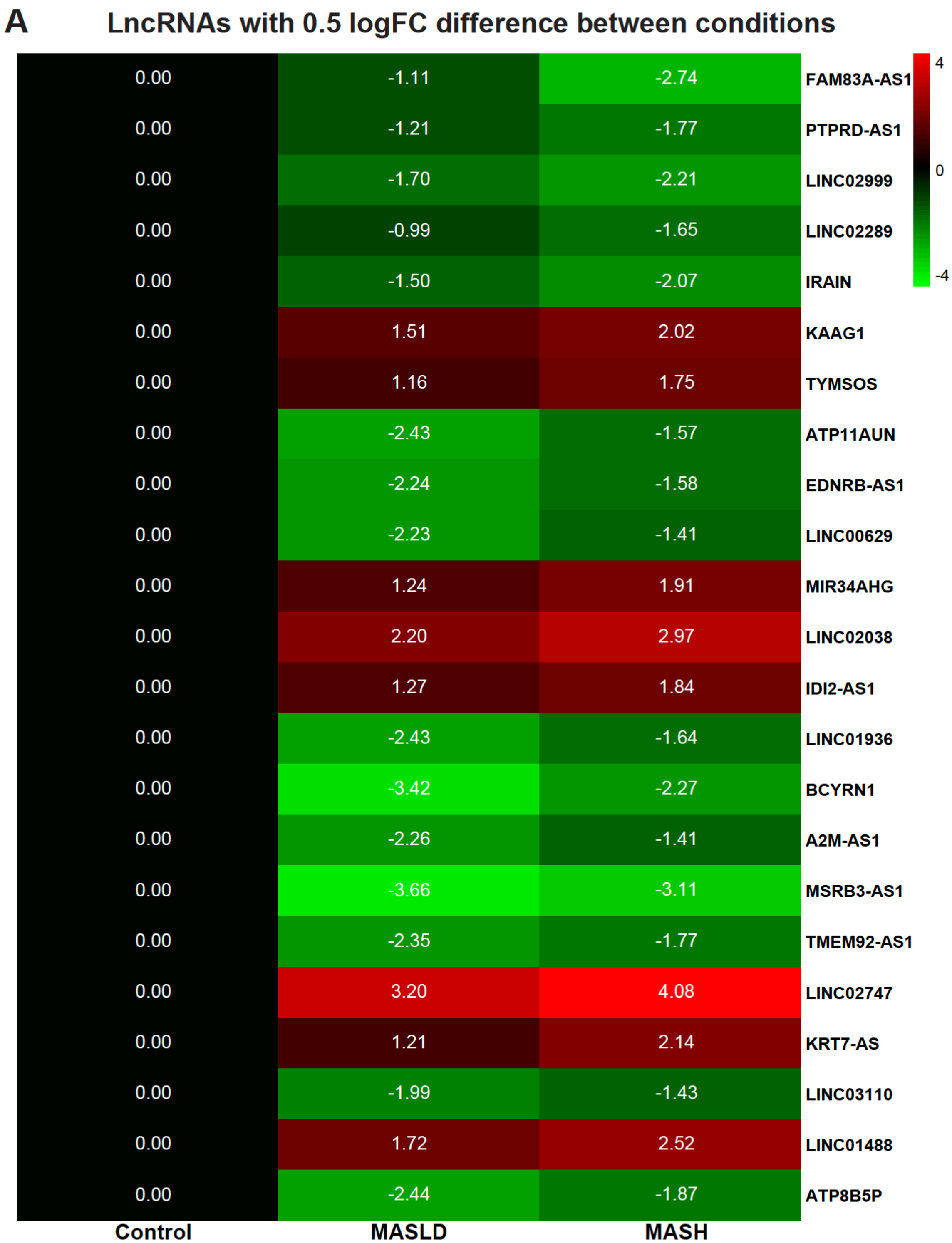

Supplemental Figure 1. Identification of a 23-lncRNA gene set signature. The expression patterns of **s**tatistically significant lncRNAs (padj < 0.05) that demonstrated a 0.5 or more log2(fold change) difference between the MASLD and MASH conditions are illustrated using a heatmap. A log2(fold change) scale ranging from 4 to -4 is included, which indicates the up-regulated (red) and down-regulated (green) regions in the dataset.

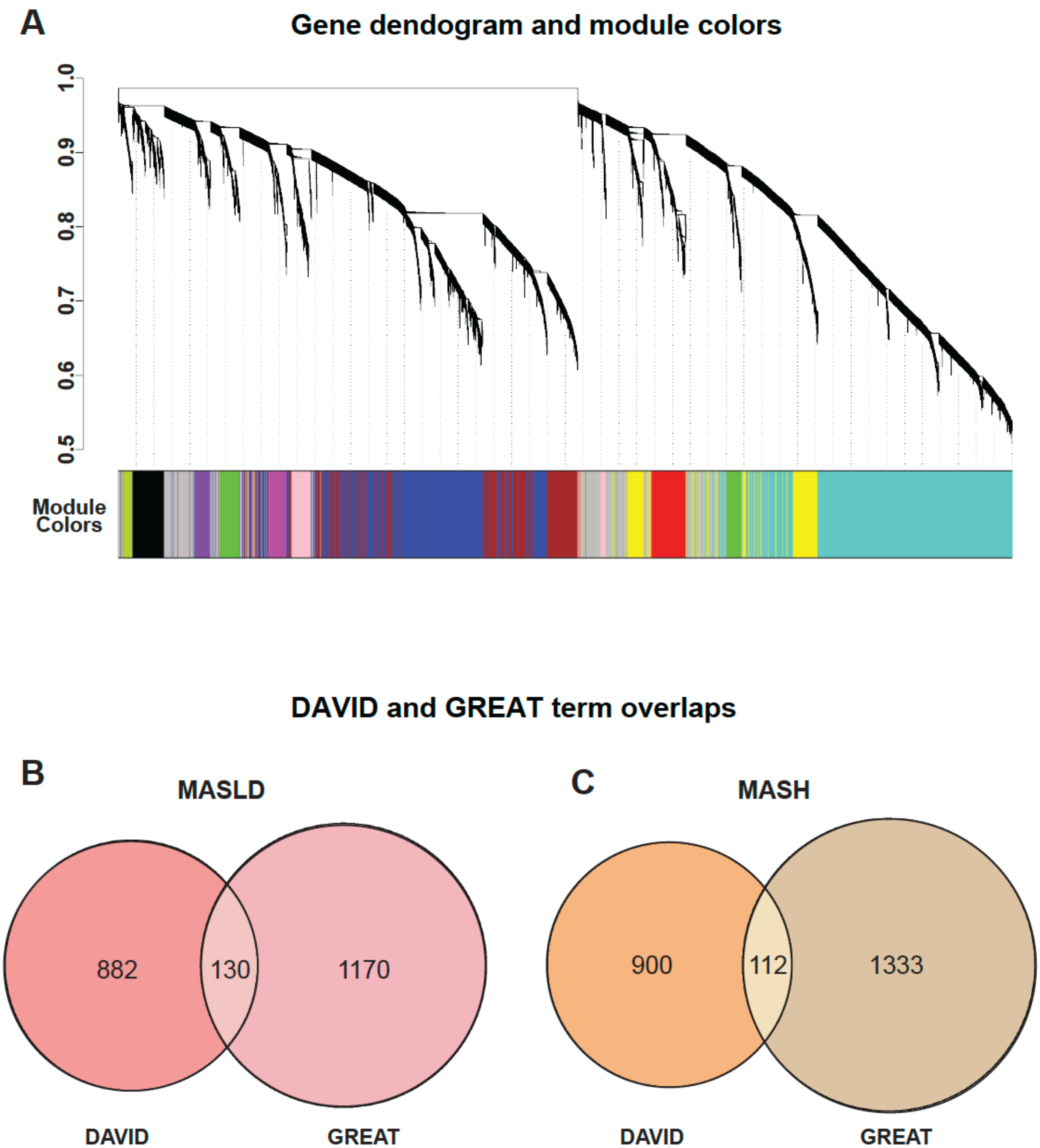

Supplemental Figure 2. WGCNA of protein and non-coding genes provides reinforcement of GREAT GO terms. (**A**) Hierarchical cluster tree showing the 12 modules of co-expressed genes identified in the GSE135251 dataset. The lower panel of the graph depicts all modules, which are marked by specific colors, such as 'MEturquoise' and 'MEblue'. Co-expressed genes in statistically significant modules were submitted to DAVID to obtain GO terms. DAVID GO terms were compared to GREAT terms for all DEG lncRNAs in both sample types. Intersection Venn diagrams illustrate the DAVID and GREAT term overlaps in (**B**) MASLD and (**C**) MASH.

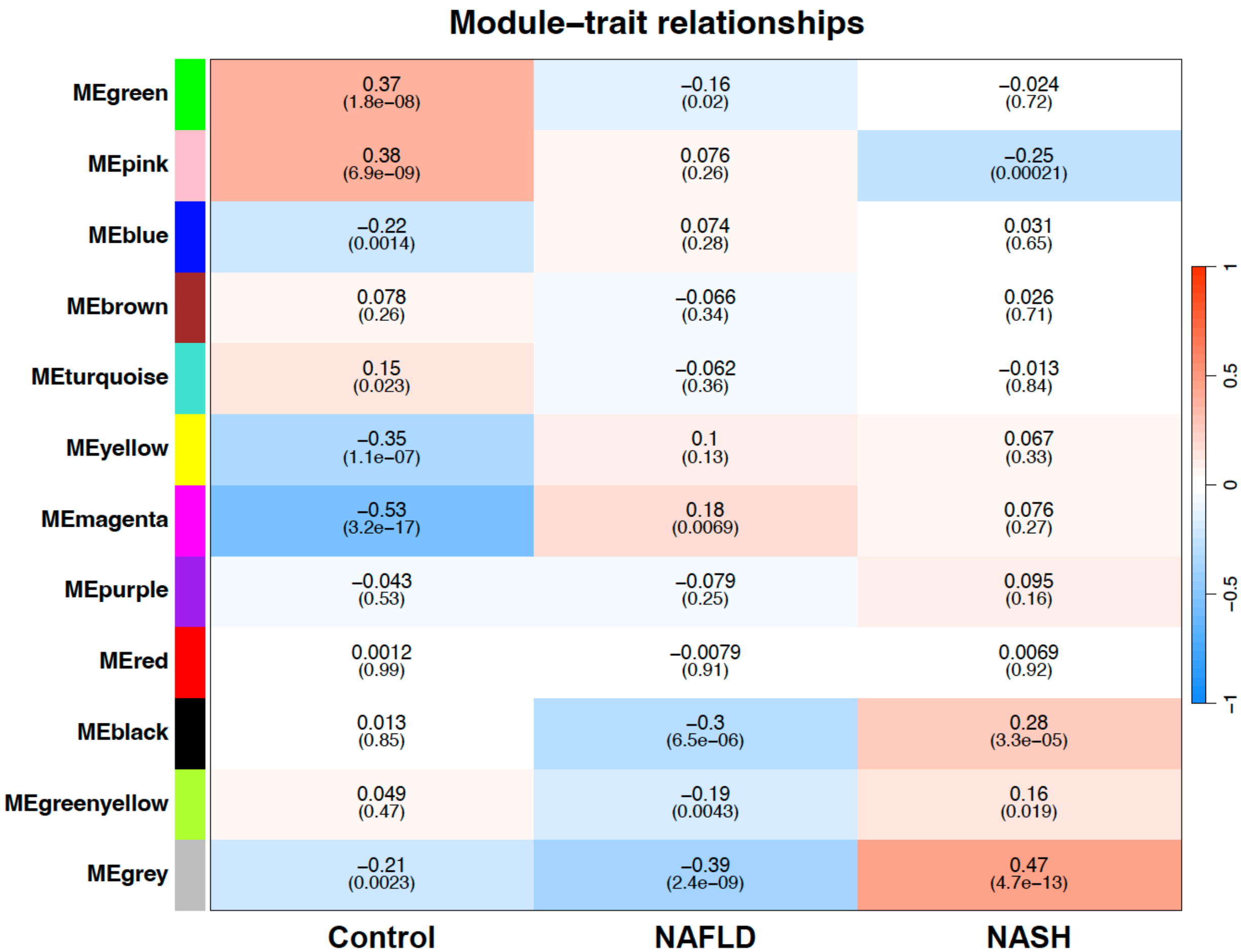

Supplemental Figure 3. WGCNA Module-trait relationship analysis of protein and non-coding genes. Heatmap represents the module-trait relationship of the WGCNA analysis. The color scale on -the right shows module-trait correlation from –1 (blue) to 1 (red), with corresponding P-values included in parentheses.

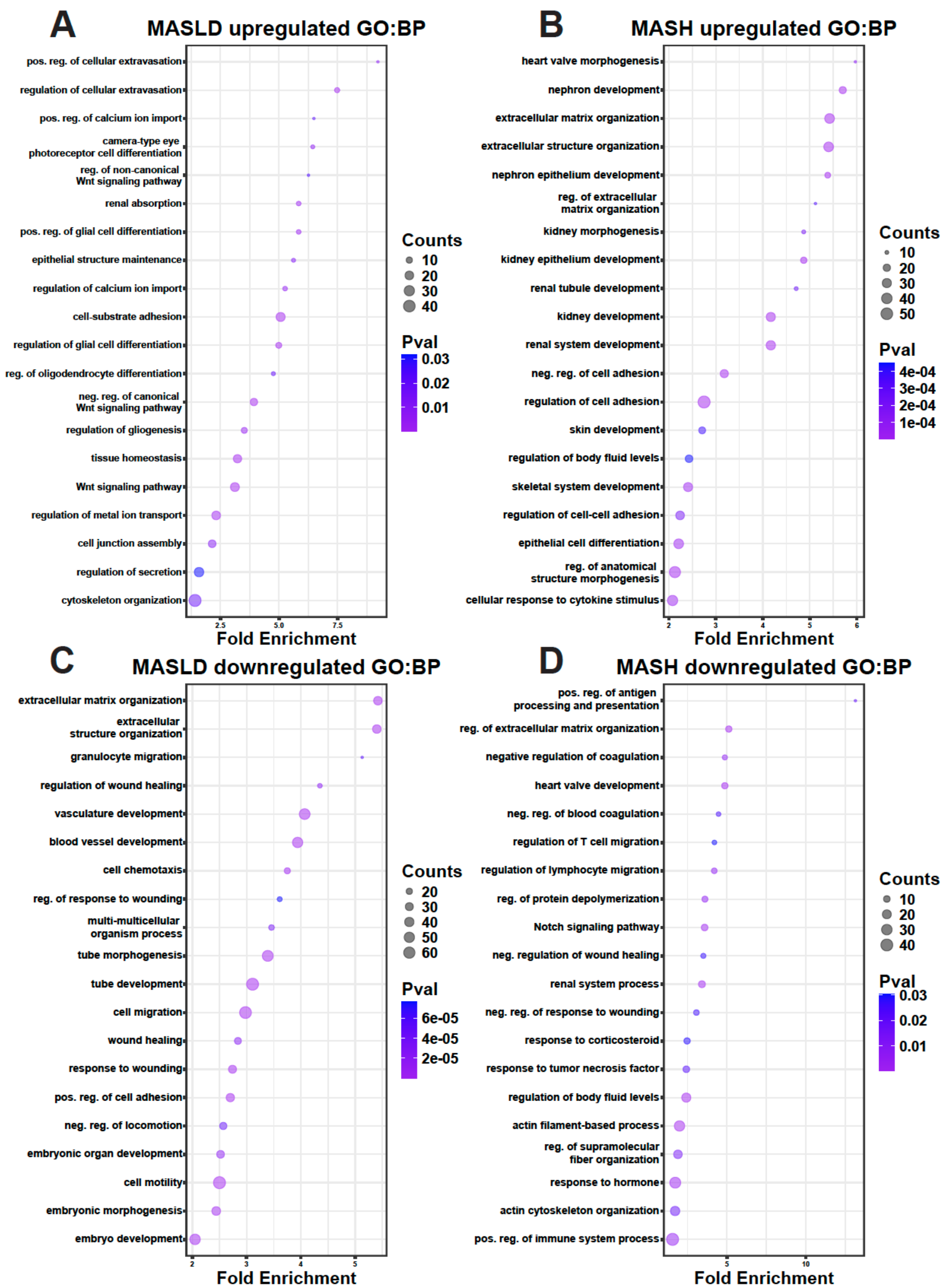

Supplemental Figure 4. Biological Processes Gene Ontology overlapping terms from DAVID and GREAT analyses. Co-expressed genes in statistically significant modules were submitted to DAVID to obtain Biological Processes GO terms. These terms were compared to GREAT Biological Processes terms for (**A**) lncRNAs up-regulated in MASLD; (**B**) lncRNAs up-regulated in MASH; (**C**) lncRNAs down-regulated in MASLD; (**D**) lncRNAs down-regulated in MASH, to identify overlapping terms.

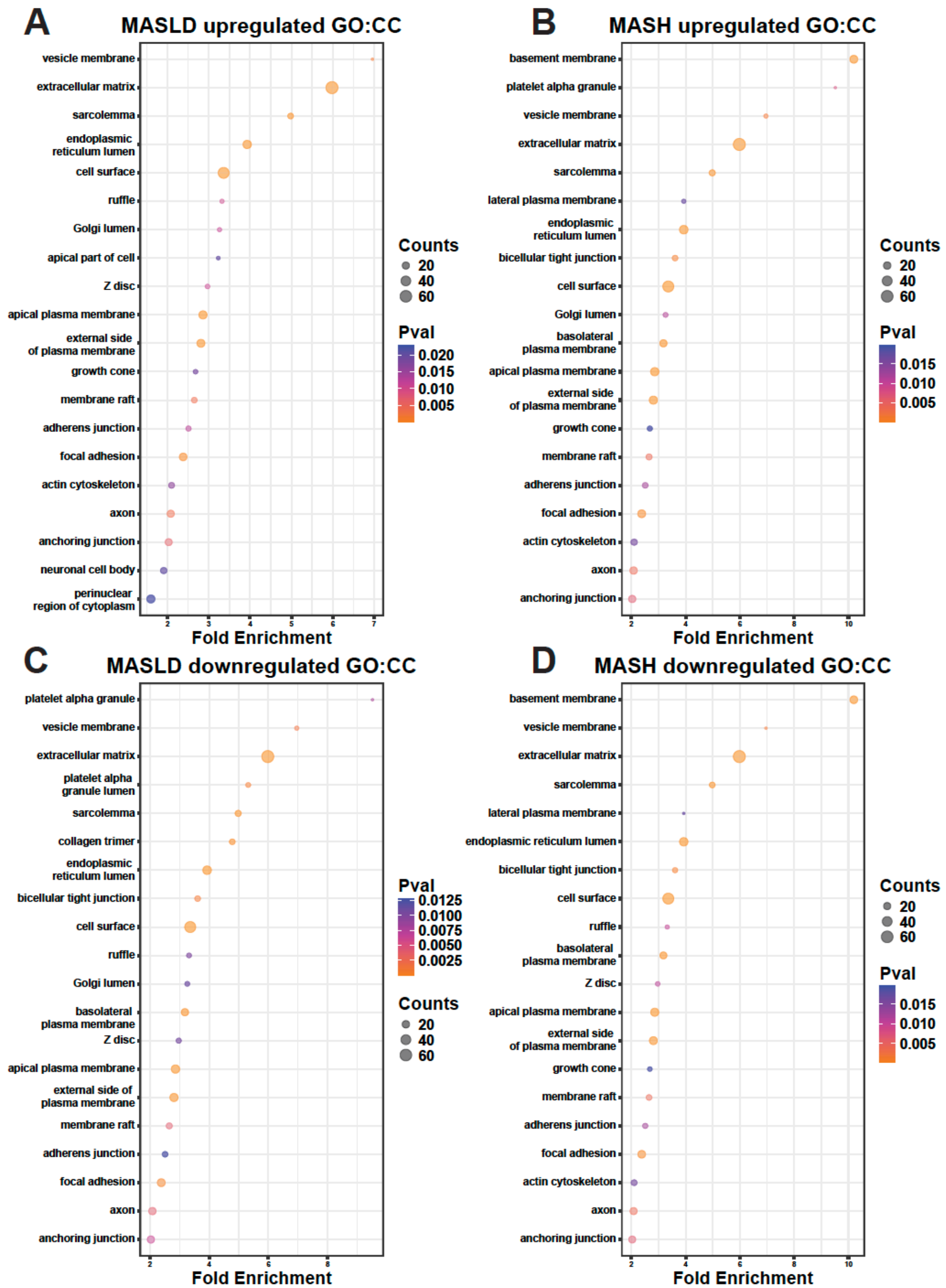

Supplemental Figure 5. Cellular Component Gene Ontology overlapping terms from DAVID and GREAT analyses. Co-expressed genes in statistically significant modules were submitted to DAVID to obtain Cellular Component GO terms. These terms were compared to GREAT Cellular Component terms for (**A**) lncRNAs up-regulated in MASLD; (**B**) lncRNAs up-regulated in MASH; (**C**) lncRNAs down-regulated in MASLD; (**D**) lncRNAs down-regulated in MASH, to identify overlapping terms.

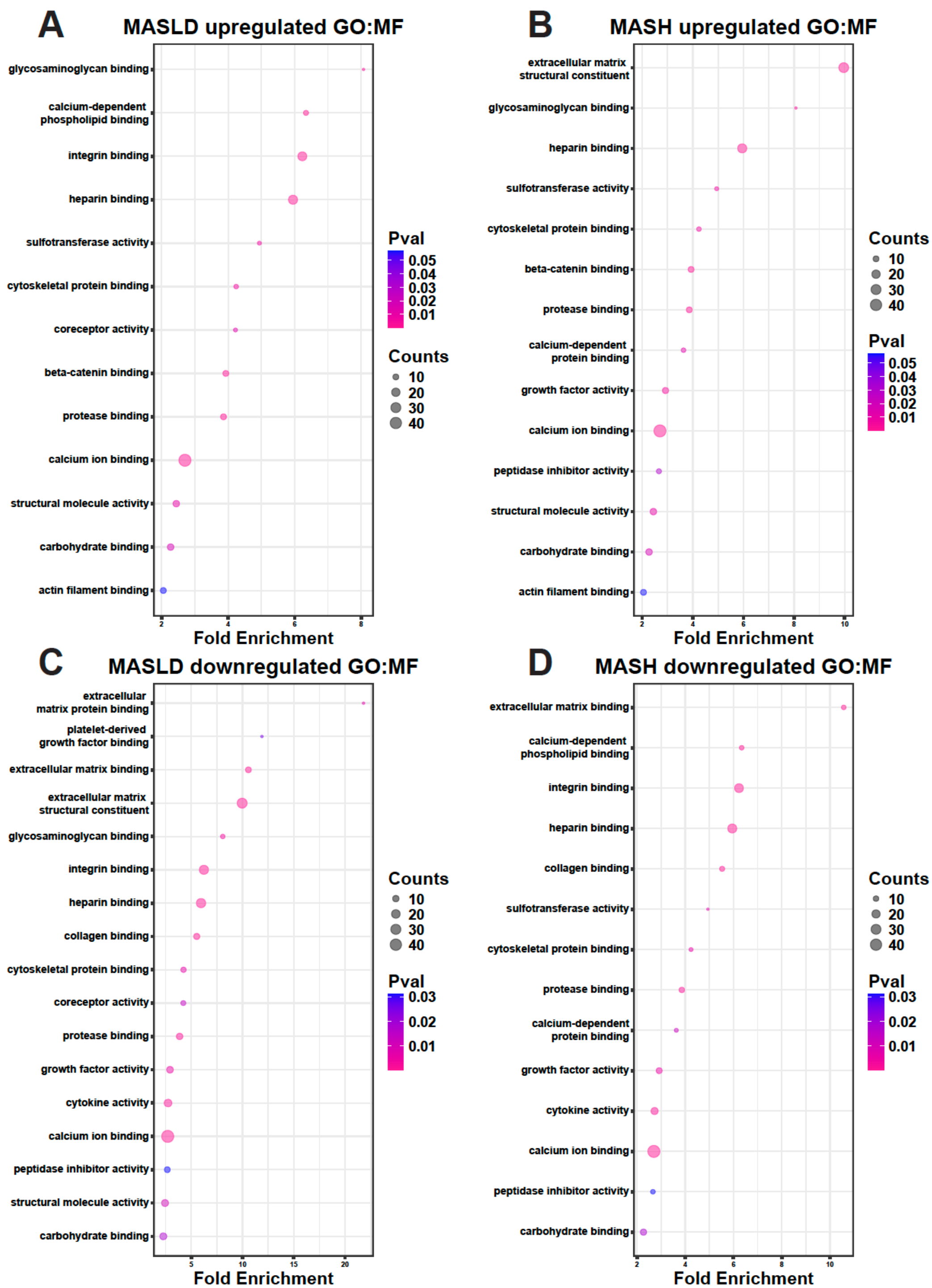

Supplemental Figure 6. Molecular Function Gene Ontology overlapping terms from DAVID and GREAT analyses. Co-expressed genes in statistically significant modules were submitted to DAVID to obtain Molecular Function GO terms. These terms were compared to GREAT Molecular Function terms for (**A**) lncRNAs up-regulated in MASLD; (**B**) lncRNAs up-regulated in MASH; (**C**) lncRNAs down-regulated in MASLD; (**D**) lncRNAs down-regulated in MASH, to identify overlapping terms.

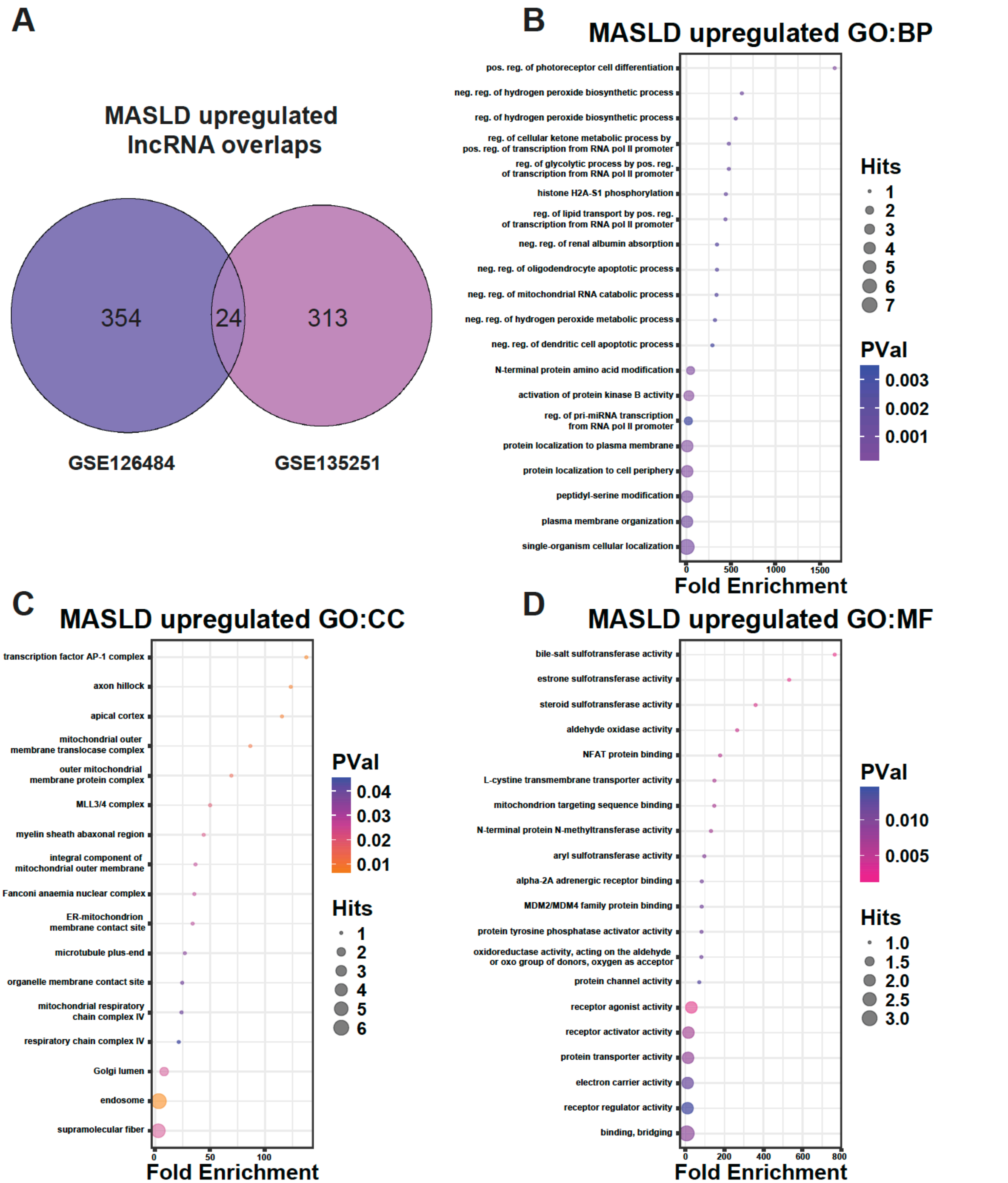

Supplemental Figure 7. Identification and function prediction of upregulated MASLD lncRNAs across two datasets. Up-regulated lncRNA gene lists in MASLD samples were obtained from both the principal (GSE135251) and validation dataset (GSE126848). (A) Intersection Venn diagrams illustrate the lncRNA overlaps between both datasets. GREAT analysis was employed to predict the functions of these overlapping genes for (B) biological processes, (C) cellular components, and (D) molecular functions.

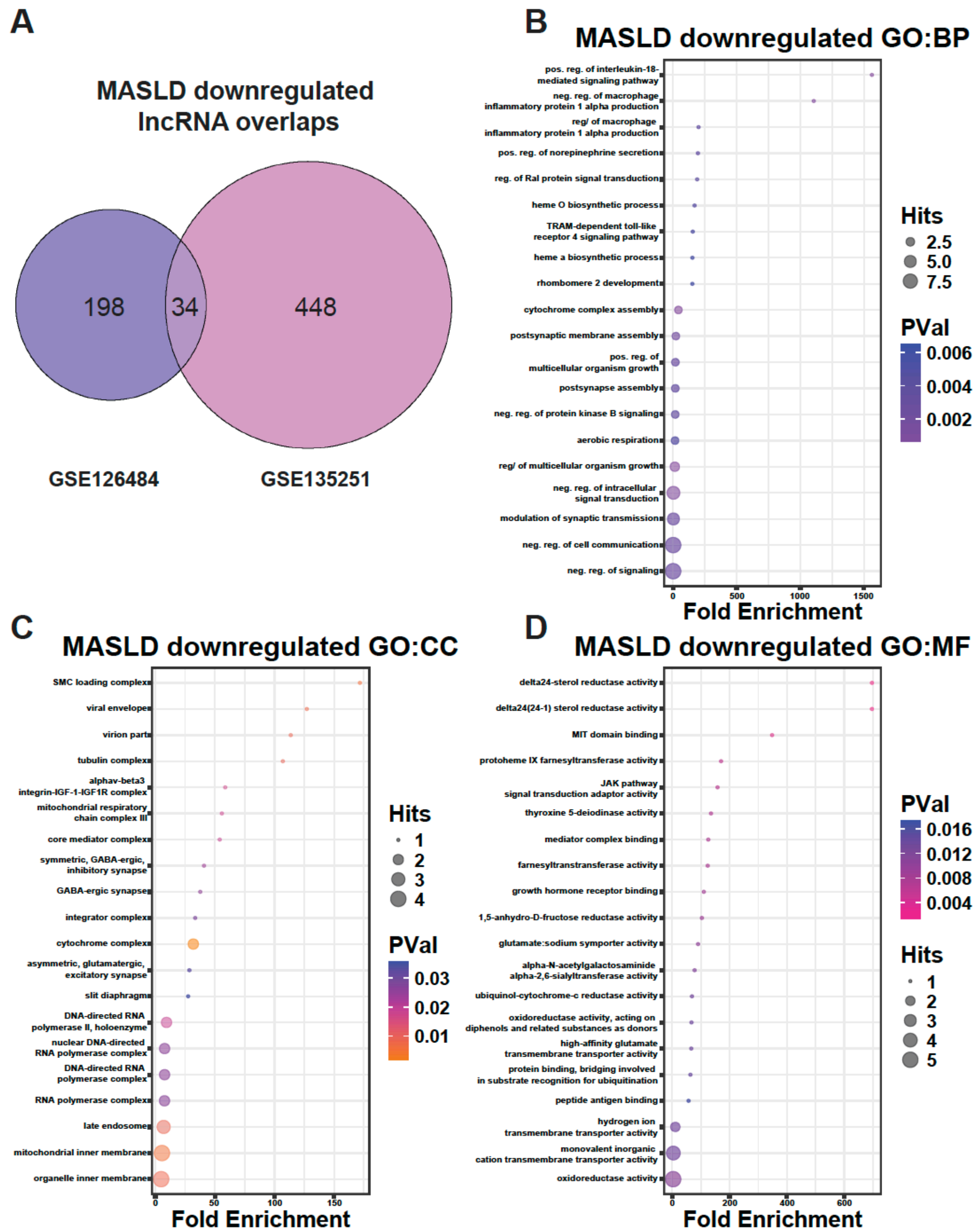

Supplemental Figure 8. Identification and function prediction of downregulated MASLD lncRNAs across two datasets. Down-regulated lncRNA gene lists in MASLD samples were obtained from both the principal (GSE135251) and validation dataset (GSE126848). (A) Intersection Venn diagrams illustrate the lncRNA overlaps between both datasets GREAT analysis was employed to predict the functions of these overlapping genes for (B) biological processes, (C) cellular components, and (D) molecular functions.

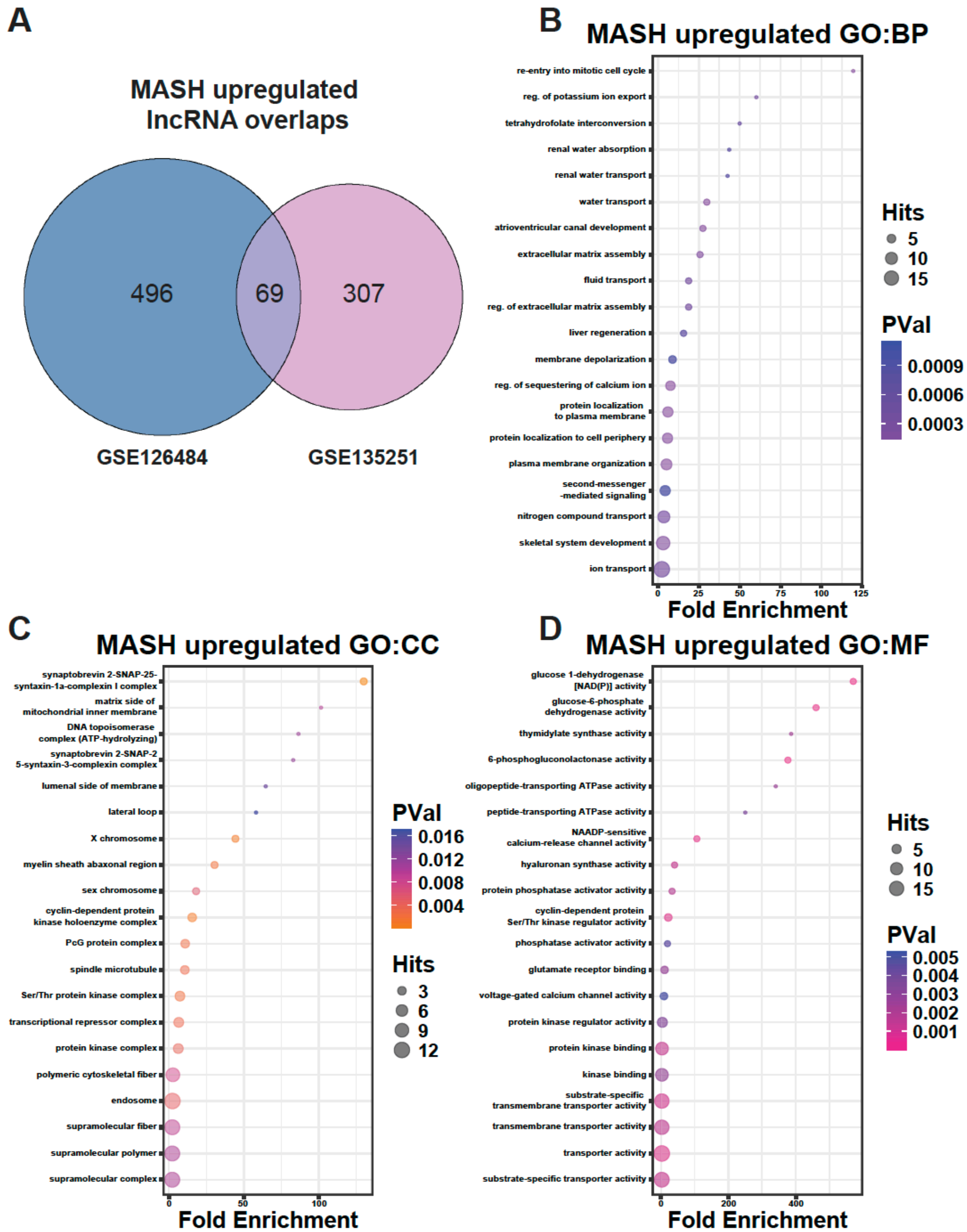

Supplemental Figure 9. Identification and function prediction of upregulated MASH lncRNAs across two datasets. Up-regulated lncRNA gene lists in MASH samples were obtained from both the principal (GSE135251) and validation dataset (GSE126848). (A) Intersection Venn diagrams illustrate the lncRNA overlaps between both datasets. GREAT analysis was employed to predict the functions of these overlapping genes for (B) biological processes, (C) cellular components, and (D) molecular functions.

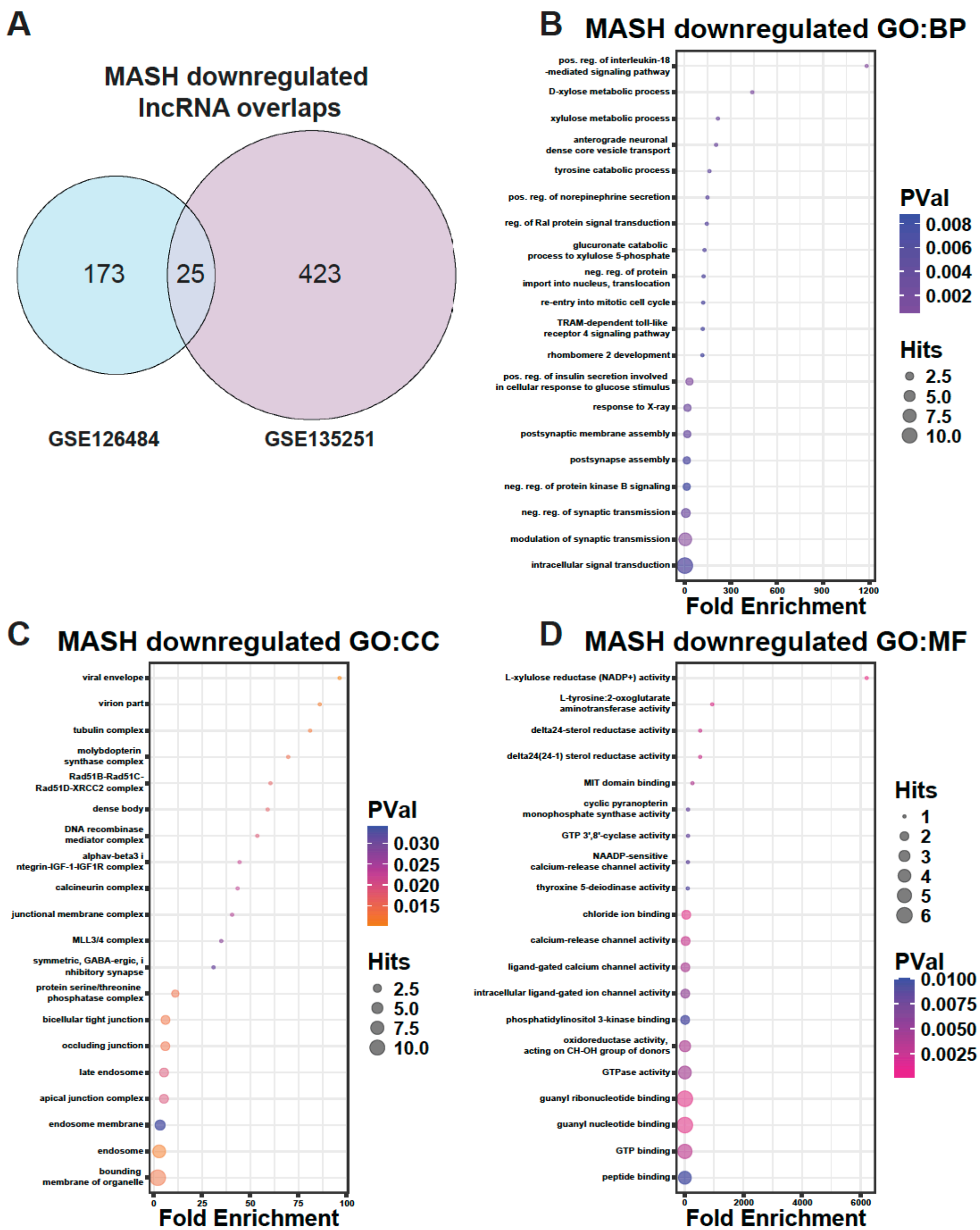

Supplemental Figure 10. Identification and function prediction of downregulated MASH lncRNAs across two datasets. Down-regulated lncRNA gene lists in MASLD samples were obtained from both the principal (GSE135251) and validation dataset (GSE126848). (A) Intersection Venn diagrams illustrate the lncRNA overlaps between both datasets GREAT analysis was employed to predict the functions of these overlapping genes for (B) biological processes, (C) cellular components, and (D) molecular functions.
